# Supplementary figures and images for: Coral spawning patterns in the Gulf of Thailand reveal synchronised annual daytime spawning, with a review of spawning patterns in Pavona corals across the Indo-Pacific
Source: PLoS One. 2026 Feb 20;21(2):e0343346. doi: 10.1371/journal.pone.0343346 (PMC12922984; doi:10.1371/journal.pone.0343346)

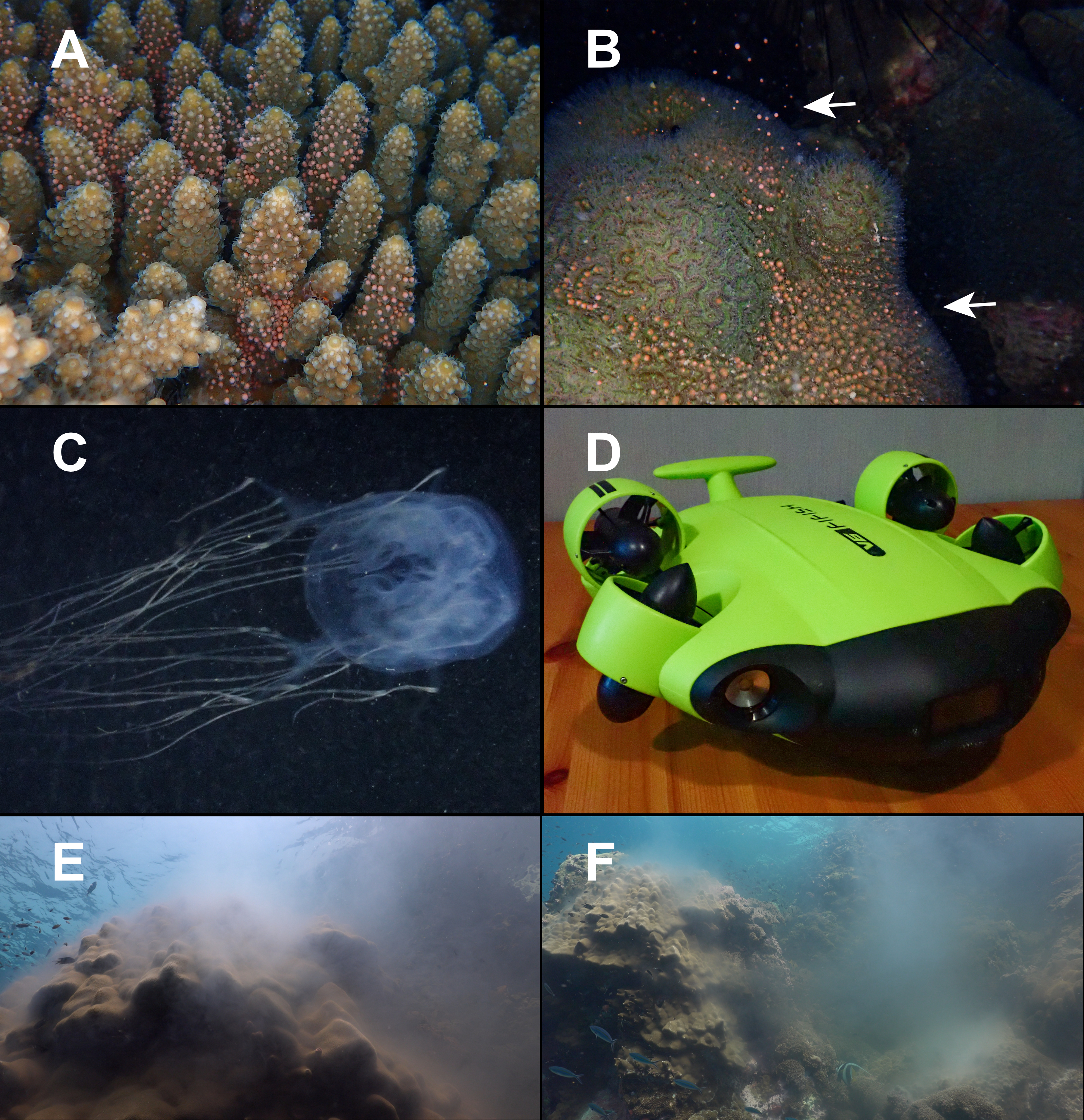

Supplement: S2 Fig — (JPEG) [file pone.0343346.s002.jpeg]

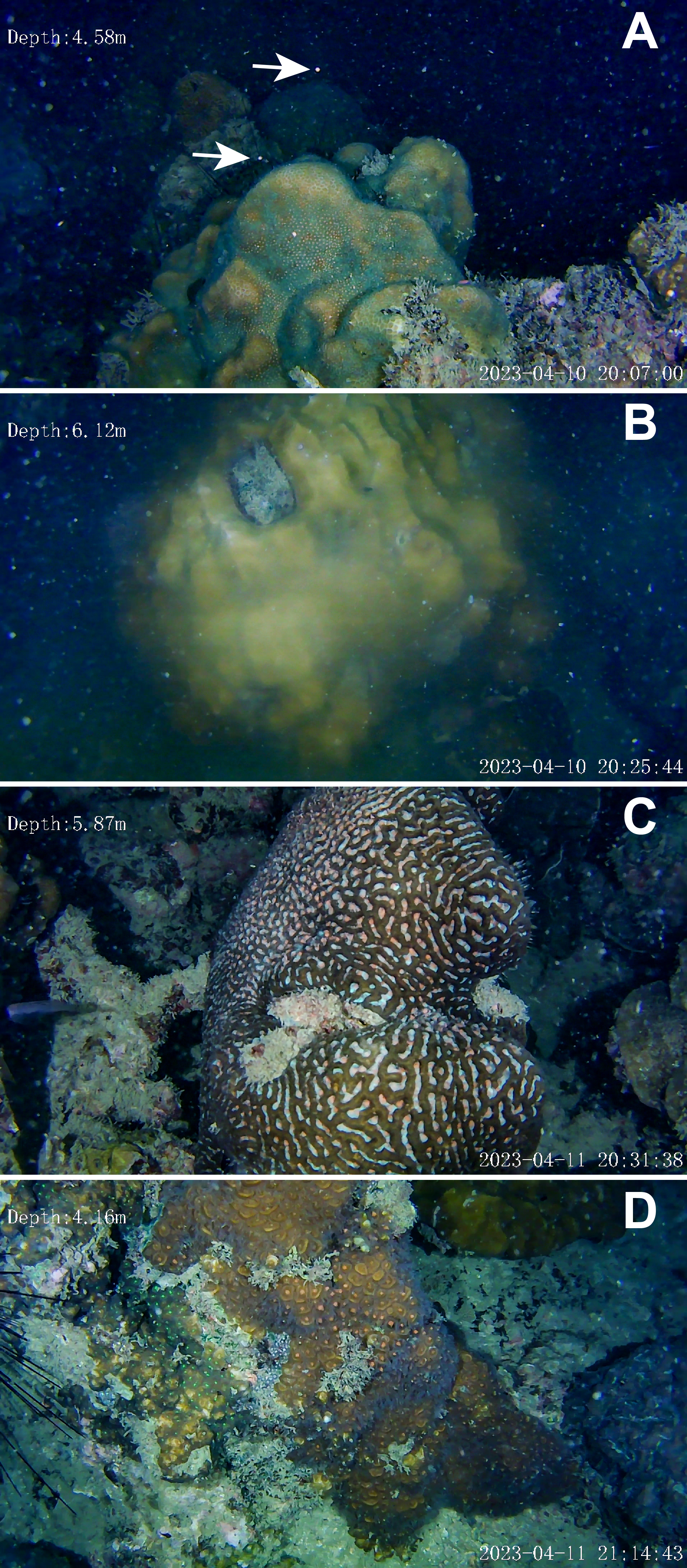

Supplement: S3 Fig — Gamete bundle release of Goniastrea, example of released bundles shown by arrows (A), sperm release by Porites (B), setting of gamete bundles prior to spawning in Platygyra (C) and Favites (D). Scale bars not provided due to lack of scaling tools associated with ROV. (JPEG) [file pone.0343346.s003.jpeg]

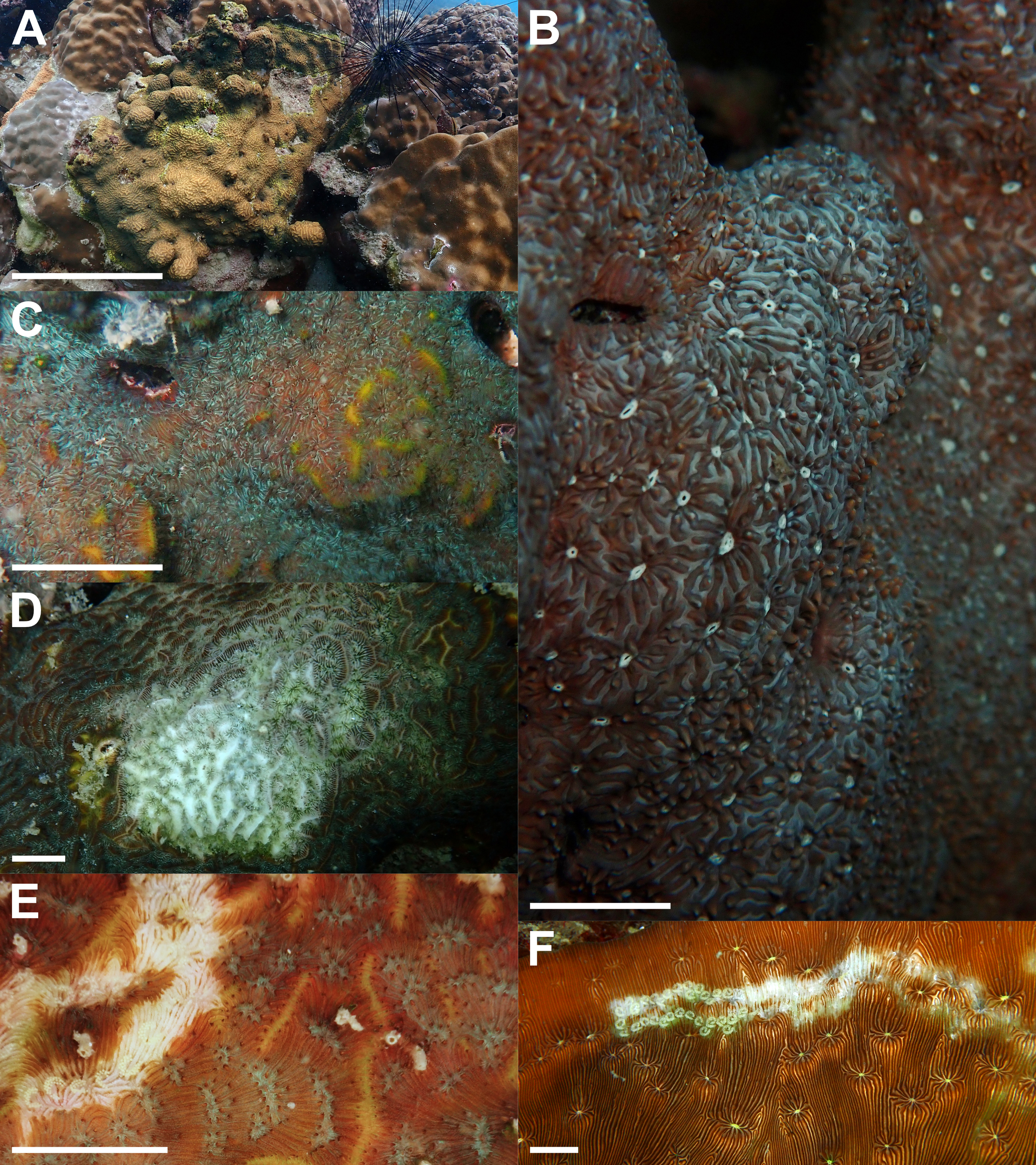

Supplement: S4 Fig — (JPG) [file pone.0343346.s004.jpg]
