# Supplementary material for: Coral spawning patterns in the Gulf of Thailand reveal synchronised annual daytime spawning, with a review of spawning patterns in Pavona corals across the Indo-Pacific
Source: PLoS One. 2026 Feb 20;21(2):e0343346. doi: 10.1371/journal.pone.0343346 (PMC12922984; doi:10.1371/journal.pone.0343346)
Supplement: Attachment — Submitted filename: Responses to Reviewers Comments.pdf [file pone.0343346.s007.pdf]

Reviewer #1:

The paper presents relevant information on coral spawning in an understudied location, and is generally well-written. Although I recognize the significance of this research, I have one major issue with the paper and this is about the inclusion of null data, specifically with the taxon (see Table S1). It is crucial to identify the spawning organism, and entering null data renders it untrustworthy. I would advise excluding these data unless the authors were able to identify them, even at the lower taxonomic level (e.g. family). Additionally, there are a lot of null data for the taxon (396, more than 50% of the listed taxon), which raises doubts about whether the observations are actually from hard corals or from other taxa. I have chosen to reject this paper for the time being because this information is the foundation of this study. I do, however, advise the authors to submit again after the identification of the species have been resolved. I also added some more suggestions/clarifications below.
Title: I feel that the title is misleading. You also discussed about other spawning coral species and night time spawning in the gulf and not only Pavona, therefore, I suggest revising this title appropriately.

- **Authors Response**: We greatly appreciate the reviewer's efforts to improve the quality of our work and the detailed comments made. We concede the reviewer's point that this paper does indeed talk about other spawning corals. We note however:
    - There is a clear and distinct emphasis on *Pavona* corals on the majority of figures, tables, and results of our work.
    - Other spawning corals are included for context, and partially draw on previously published literature.

  Nonetheless, we have reworded the title to be more inclusive, while retaining the clear emphasis of our work. We have responded to the reviewer's questions and confusion regarding the methodology, including use of null data, among other aspects in the responses below. The lack of null data is a well-known issue in identifying spawning periods, and we acknowledge that the null data cannot confirm absence and presence of every single coral taxa evenly per survey. What it absolute does do is narrow windows of possible spawning periods, as incidental observations of spawning are a) precisely how initial patterns for this project were found and b) reported with sufficient frequency and reliability to be shared as valid novel findings. We are optimistic that the reviewer's concerns are adequately addressed below.

Abstract
Line 30: Looking at your data (Table S1 and Fig. 4), it does not seem to be synchronous spawning across the gulf but to certain locations and to some colonies. For example,

Pavona explanulata spawned on June 15-16 and 18-19, 2024 at Koh Koun, on June 16, 2024 at Hin Yuan, on June 17, 2024 at Koh Rong Sanloem. In 2022, it spawned on October 9 at Hin Yuan, October 11 at Hin Ploeng, November 9 at Hin Yuan and Koh Koun. Please revise accordingly.

- **Authors Response**: We appreciate the reviewer's concern regarding our assessment of synchrony, however it appears the reviewer has perhaps misread the aforementioned figure and table. Neither the table nor the figure show any spawning in June (which could not have been recorded anyway as this was during the inaccessible monsoon period, as mentioned in the methodology). To improve clarification, we have updated table S1 to avoid confusion regarding the date format across the table, however these may easily be cross-referenced with the original table accessible to the reviewer for any further clarification needed. Regarding Figure 4, we remind the reviewer that the x axis begins at the month of August and thus cannot show data from June. We have, however, updated our caption for Fig. 4 to improve clarity on this point and we appreciate the reviewer demonstrating the need for this. Finally, the majority of our paper discusses and references the role of lunar, solar, and other environmental variables on the synchrony of coral spawning. Regarding the reviewer's concerns regarding the offset of days and months in the synchrony of spawning in our data, we note that, in many (but we concede not all) cases where surveys were indeed carried out concurrently, synchrony of spawning was absolutely observed across the provinces and between the countries. This re-emphasises the value of the null data (where available) such that readers and reviewers may see where surveys were carried out but spawning was explicitly not seen, versus an absence of data due to lack of a survey. Secondly, synchrony within the coral spawning literature does not only apply to within a window of minutes/hours, but spawning events may also be considered synchronous if they spawn across the same spawning period (multiple colonies, per night, within the hours/days preceding or following the full moon or new moon). The offset across multiple months is entirely normal within coral spawning literature, and does not indicate a lack of synchrony, just that spawning events may be (and typically do) spread across more than one lunar cycle (most months typically only having a single lunar peak and so on). A lack of synchronisation is noted when the same environmental and biological cues no longer apply to observed spawning, which we also make some effort to discuss in our work.

Introduction

Line 89: Please cite these few studies here.

- **Authors Response**: We have moved the sentences that cite this work to the relevant section of the paragraph and have divided the remainder to improve the follow of the introduction for the reader.

Line 97-100 I would use caution while making this claim and advise the authors to reword it. Removing an entire colony from the reef is not less destructive.

- **Authors Response**: The reviewer's opinion that a temporary removal and return of a coral colony from the reef is not less destructive than permanent removal of often large sections of a colony, is an entirely fair one. While the authors' disagree with this opinion, it does not change that fact that the second word used in the sentence is ''potentially'', to avoid framing in absolute terms, and is based upon communications with local practitioners who do consider the practice ''less destructive''. We also do not necessarily agree with the opinions of these practitioners, instead framing the practices as distinct and having it's own nuances. In this section, we have not established the details nor generalised the a) size of colonies, b) size of fragments, c) short-term vs. long-term costs vs. benefits, etc. The purpose of this paragraph is to present context and concepts for the practices that have been and often are being conducted, that are relevant to our survey methodology as well as our study sites as a whole. Given that we have not only cited references, but also used an abundance of caution in our framing, (e.g. the following sentence explicitly raises concerns on the potential damage of this practice, aligning more with the reviewer's perspective), we have not removed nor reworded this section).

Lines 90-101: Rather than dedicating this whole paragraph by explaining the various methods of coral reproduction, I suggest that this should be rewritten focusing on mass coral spawning in Thailand. In fact, while reading Line 89, I have expected that the next sentences would be about mass spawning or coral reproduction in the Gulf of Thailand.

- **Authors Response**: We have worked to improve the readability of this section and moved the concluding sentences and citations that specifically talk about spawning observations to the follow the start of this paragraph, with a natural lead into the gamete-development assessments and the relevant survey methods. We note however that this paragraph does not talk about ''various methods of coral reproduction'' but rather the methods used by surveyors to assess this and predict coral spawning timing. We hope the clearer distinction between the earlier spawning records (the entirety of which have been stated in the appropriate line) and the techniques used to determine reproductive period.

Results

Line 201-202: Did all spawning occur in all years of observation? If not, please add the years here when spawning was observed.

- **Authors Response**: Indeed, spawning was observed in all surveyed years. We have amended this section to clarify this for readers.

Line 248: Suggesting that these are brooding over broadcast spawning is an overspeculation and should not be written in results. Perhaps you can mention this in the discussion with an added rationale. Also, sperm spawning may suggest gonochoric sexuality but not reproductive mode.

- **Authors Response**: We appreciate the reviewer's suggestion on this point, and while the entirety of this part of the results explicitly explores the differences between gonochoric and hermaphroditic sexuality, the hypothesising of reproductive mode is best explored in the Discussion if needed. We have thus removed the wording "possibly indicating brooding over broadcasting".

Line 259-260: This is discussion material and should be removed from here.

Authors Response: We appreciate the reviewer's suggestion, we have removed the indicated sentence from the Results section.

Table S1: I highly recommend that null data for the taxon level should be replaced with species identity even in the lowest possible resolution, for instance family.

Authors Response: Unfortunately, this is not possible and would reduce the value of our work, due to the methodology employed (clarified above). The surveys carried out across the reefs, including our night-time surveys and all surveys where spawning *was* documented, incorporated all corals and other organisms in the area where possible. Null data there explicitly indicates an absence of any spawning from any observed corals. While we can appreciate that this does not easily allow the reviewer to note which corals were not found to be spawning, we have amended our manuscript with multiple references to our other published material that more directly and comprehensively cover the coral community composition of the sites surveyed. A repeat of this data would not be able to fit in the supplementary table, and would largely amount to a direct duplication of the same data shared from earlier work. Instead, we have opted to *discuss* the absence/presence of spawning corals, in direct reference to this.

=Suggest revising Notes to “Notes or Reference/s”

- **Authors Response**: Thank you for this suggestion, we have made this edit.

=Be consistent with the formatting of your dates.

- **Authors Response**: Thank you, we have reviewed the table and made this amendment throughout.

=Please italicize species name. Also check species name where the specific epithet has been capitalized (e.g. Pavona Explanulata).

- **Authors Response**: Thank you, we have reviewed the table and made this amendment throughout.

= I would recommend restructuring this table for better organization, perhaps arranged by species, or date of observations.

- **Authors Response**: We appreciate the reviewer’s recommendation and have made numerous edits to allow the data to be filtered in numerous ways. However, the overall order of observations remains as presented (with each record having a specific number) such that cataloguing and review as per our data organisation is maintained. We trust that readers will have the capacity to use the in-built data filtration system to prioritise organisation based on specific needs.

= Should be “Table S1. Coral spawning records observed along the Eastern Gulf of Thailand during the five-year survey period (2020-2024), compiled alongside published records of Pavona spawning from across the Indo-Pacific”

- **Authors Response**: We have amended this based on the reviewer’s suggestion, with the addition of ‘’ ...compiled alongside published records of prior spawning in the Gulf of Thailand and *Pavona* spawning from across the Indo-Pacific”

Figure 2: Do you have a better photo of c? Current photo is blurry or perhaps its just my copy.

- **Authors Response**: Unfortunately, we are faced with two challenges here in assisting the reviewer’s entirely valid concern. Firstly, it is quite likely that the Figure images made available to reviewers have undergone the usual heavy compression from submission of raw image files. Therefore, in an effort to allow the reviewer to properly

assess the resolution of our figures, we have included them, with captions, at the end of our responses to reviewers. We have also copied a snapshot of the relevant part of our updated Figure 2 in this response to more easily allow for further review. Secondly, the egg release in P. explanulata in our observations was highly challenging to capture clearly due to the size of the gametes, form of release, as well as the lack of distinctive pigmentation allowing for sufficient contrast. To aid in visualisation, we have amended this figure (and caption) to more clearly indicate the release of eggs.

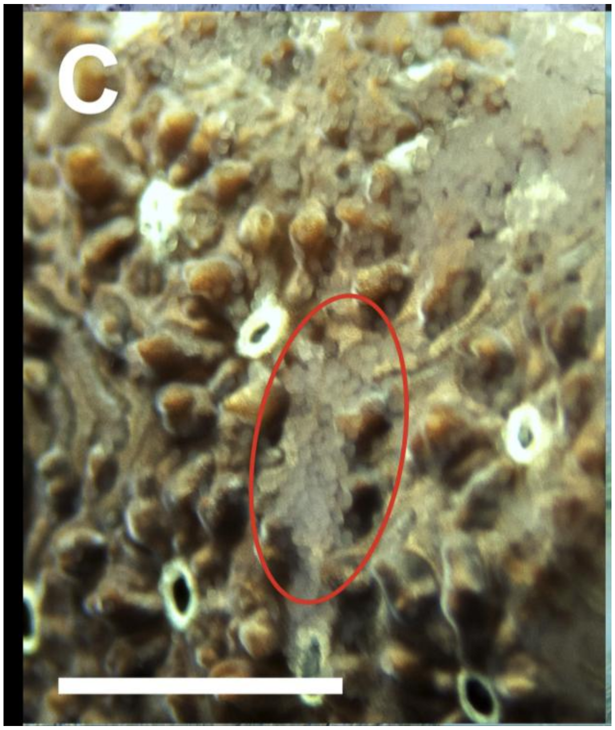

Figure 3: I think it's inappropriate to include night time spawners in this figure if you aim to use sunrise as reference of spawning. Perhaps divide figures for daytime and night time spawners (this using hours after sunset).
Figure 5: Same comments with Fig. 3

- **Authors Response** (Fig. 3 and 5): We appreciate the reviewer's concern that the hours after sunrise may not be applicable for night time spawning. However, the reviewer's assumption that sunrise timing does not influence nighttime spawning is unfounded and unsupported by the literature (as discussed in our paper). Hours after sunset has historically been the framing for spawning timing, however there is no evidence to suggest that this in any way adds more accuracy or relevance than sunrise. We find that complicating visualization into multiple figures purely to follow historical convention does not add value. Adherence to a historical lens to view coral spawning adds a

historic bias, and we instead here find value in re-visualising all spawning through a different lens without changing the data itself. It is no less convenient than using sunset time, and indeed our data suggests that sunrise time may indeed be a more appropriate, if not more precise, cue to the onset of spawning. Of course, this particular hypothesis needs considerably more work to validate, but is nonetheless a valid framing of our data. Therefore, in lieu of greater justification than subjective convenience to the reviewer, we have left these axes and figures unchanged beyond font size and readability.

Discussion
Line 310: Be careful with the use of synchronous mass spawning. Your results did not show synchronous spawning if you look closely. Spawning 784 colonies and previous studies do not occur at a brief period of time, but across months and years. Indeed, you documented various spawning events with a number of colonies and species spawning at around the same time. But the overall pattern did not show synchrony.

- **Authors Response**: As per our earlier response, this comment is unfortunately reflected by the reviewer's misunderstanding of the data and visualisations provided (see response re: Table S1 and Fig. 4 above). As a result, we have attempted to make numerous modifications throughout this work to more easily allow readers to navigate the scale and nuances of the data provided. Again, we believe we have demonstrated with a high credence that multiple colonies spawning within minutes to hours of each other, within a single site, across sites, and seemingly across nations, over multiple years, and seemingly at no other times (though we have already eluded to margins of error) does leave room for little doubt that synchrony is absolutely present. Given this point being a somewhat subjective disagreement of opinion and interpretation based purely on data, and our conclusion (and central premise) of mass synchronisation, we have not made changes to this section.

Line 311: You did not determine development and measure reproductive output in this study so I recommend removing this part.

- **Authors Response**: We however did measure reproductive timing and absolutely did quantify reproductive output in the number of colonies per observation (see Table S1). We have therefore amended the sentence to emphasize the *inference* as opposed to measurements of reproductive development (given we documented carefully the release of gametes and when).

Line 318-320: I strongly believe you should revise this. You did not conduct spawning observations prior to September, so the term lack of data is more appropriate than considering this previous observation as outlier.

- **Authors Response**: We appreciate the reviewer's concern regarding our framing of the sentence. We have therefore added the following sentence at the appropriate section: "However future surveys in the months of May to August (outside of the typical monsoon period for the western Gulf) may reveal trends that expand this period."
  However, the reviewer is incorrect in their statement suggesting we did not conduct spawning observations prior to September. While this study did not conduct surveys between May and August, our surveys did comprehensively include surveys from January to April (and indeed, parts of May). Additionally, we have cited our earlier work from the western Gulf of Thailand that did survey this period which have also been included in Figure 5 and related text. Our results and discussion do not avoid mention of gaps in our data (months, years, and hours between sunset and sunrise for much of the year) which we discuss freely. While the month of August specifically remains poorly surveyed, our data clearly shows that the observations from Chumphon are outliers with regards to both time of the day and lunar cycle (see Figs 4,5). Therefore, while we agree with the reviewer's suggestion to emphasise the need for more data in August, we have not rephrased our use of the word outliers, given the totality of data presented.

Line 334: You mentioned Agaraciidae and non- Agaraciidae multiple times in your manuscript, thus for better readability, perhaps it's better to add another column in your Table S1 for coral family.

- **Authors Response**: We thank the reviewer for their suggestion, we have accordingly added this column in the table to clarify the presently accepted family for each taxa mentioned.

Line 345-356: This is more like a conclusion/recommendation piece, thus I would suggest placing or incorporating this in the paragraph at the end of your discussion.

- **Authors Response**: We thank the reviewer for their suggestion. We have moved the indicated lines to the end of the discussion as a concluding paragraph.

Line 357: I suggest adding Pavona in your subheading. Additionally, some portions of this

part appeared to be too long or with some irrelevant information (e.g., Lines 363-367), related to your study

- **Authors Response**: Thank you for the suggestion, we have edited our subheading accordingly. However, the lines deemed irrelevant by reviewer are as follows: *"The authors categorised the species as an 'alternating sequential hermaphrodite' where the spawning of one sex is preceded by the other within a given colony, lunar phase, and breeding season. These observations alongside additional data on gametogenic development also suggested nearly year-round reproductive activity across the range from Costa Rica, Panama and the Galapagos Islands."*.
  Given the subheading, the species, the global, regional and local data presented and discussed, and the core theme of the reproductive activity of the taxa in question, we would ask the reviewer to specify which of these lines or ideas is irrelevant to our study. For now, the authors humbly disagree with this particular suggestion and have refrained from editing these lines.

Line 365: Please add reference/s on this.

- **Authors Response**: We have repeated the citation that this section is based on the recommended line, as suggested.

Line 374-375: It should be worth mentioning that spawning observations was not conducted in May to August as opposed to other mentioned studies.

- **Authors Response**: We appreciate the reviewer's suggestion and have added a sentence to the end of the indicated paragraph to repeat and re-emphasise this.

Line 417: correlated.

- **Authors Response**: Thank you, we have made this edit.

Line 440: Moon phases (i.e. full moon) is a recognized cue for the day/night of spawning but not on exact time (hour) of the day. Maybe perhaps you can discuss on other cues of exact time such as temperature, tidal cycles, photoperiod, etc.

- **Authors Response**: We appreciate the reviewer's suggestion and have given it much consideration. We note in response however that 'moon phases' have not been a prominent variable as spawning cue in many years, largely replaced by the number of days before and after full moon. Our work applies the same concept, but has

prioritised 1) improved precision by standardising a specific reference point of what we mean by ‘’full moon’’ and improved resolution and precision by utilising hours before and after, from which of course days before and after can be easily calculated. For ease of review, we have already included a column presenting this duration in units of days instead of hours, in the supplementary material provided. As such, we do not find added value in the discussion of our data in the proposed terms for the sake of convention (which again, we have already provided some throughout this paper). We have introduced the other cues and variables in the Introduction, and the most relevant of these are discussed at depth in this section as well as preceding and following parts of the discussion. While we understand the reviewer’s suggestion as one of further discussing our work in broad terms, we have chosen to preferentially focus in on the key variables and cues for which a) we have presented (or available) data, and b) that are the most directly linked to our work.

Line 497: needed to ascertain.

- **Authors Response**: Thank you, we have made this edit.

Reviewer #2:

General comments to the authors:

The manuscript "Daytime spawning corals in the Gulf of Thailand and a review of spawning patterns in Pavona corals across the Indo-Pacific" offers invaluable information on scleractinian coral spawning in the Gulf of Thailand over multiple years, with specific focus on daytime spawning *Pavona* species. It sheds light on taxa that are often overlooked because they do not spawn during the conventional spawning time that is after sunset. By paying attention to the daytime spawners and employing creative solutions, the authors demonstrate that the overall coral spawning periods in the Gulf are between September to December and February to April. Curation of null data also allowed for further refinement of the spawning periods in this target region. The data comparison between other regions highlights that regional differences exist for month of spawning and time of the day, but not for days relative to full moon. These findings will greatly benefit researchers in coral reproductive biology and restoration projects, and advance the overall knowledge in coral spawning. Below, I list questions and minor suggestions that may help improve the manuscript.

- **Authors Response**: We are grateful to the reviewer for their comprehensive and constructive review of our work and the suggestions provided which have unequivocally helped to improve our work

---

Specific comments and questions to the authors:

I suggest thoroughly checking for present vs past tense; e.g. line 142 "...are included in our dataset". Also suggest checking for word repetitions or tautology; e.g. line 24, suggest removing "across the globe" as it is repetitive after "Global documentation"; lines 347-348 in Discussion needs rewording "reliable coral spawning ... rely on ...". Check for consistency of a word or phrase used, e.g. "daytime" and "day-time". Some of the sentences are very long (30+ words) and I suggest those to be separated or rewritten.

- **Authors Response**: We thank the reviewer for these comments and suggestions. We have reviewed the manuscript and amended various points to maintain consistency. In some cases we have also veered away from verbosity to ease comprehension.

Absract

Lines 35-37 | Suggest working in something like ", contrasting to the February to April spawning periods for nighttime spawners" to highlight the differences between daytime and nighttime spawners.

- **Authors Response**: We appreciate the reviewer's suggestion and we have made multiple attempts to do so while keeping within the word count restrictions. However, given the focus being on the daytime spawning and *Pavona* corals specifically, we have retained the current wording as being concise and specific to these topics.

Lines 38-39 | Perhaps "consistently observed" or "consistent window" is better than saying "highly predictable", because you provide empirical evidence that they indeed spawned, though this may be preference.

- **Authors Response**: The reviewer's suggestion is noted and we have replaced the word ''highly'' with ''consistently'' as we indeed did not assess the scale of prediction. However, we've retained the use of the word ''predictable'' because it directly relates to the methodology used in this study, which relied on initial observations to predict subsequent observations based on this consistency. Subsequent to the patterns being identified, null data was used to constrain these windows.

Introduction

Lines 47-51 | Suggest rewriting these two sentences as they are both currently long and difficult to read. For example, "Scleractinian corals reproduce asexually and sexually. Asexual reproduction occurs due to fragmentation and results in colony formation and growth. Sexual reproduction contributes to population turnover and adaptation through natural selection, and happens either externally (broadcasting) or internally (brooding). Sexual reproduction by both external and internal means rely on the release of sperm and hydrodynamics to disperse gametes across the reef."

- **Authors Response**: We appreciate the reviewer's suggested rewording of these sentences, which we have replaced accordingly.

Line 55 | This sentence is misleading, as "over 100 species…all spawning in a single night" is not what Babcock et al. 1986 showed. Please clarify.

- **Authors Response**: We thank the reviewer for pointing this out, we have reworded this to align with the reality of the results in Babcock et al., 1986: "Mass spawning events can involve dozens of species of corals in a given reef in a single night, with over 100 species spawning in a spawning season".

Line 66 | Remove "obvious"

- **Authors Response**: Thank you, we have made this edit.

Line 74 | What do you mean by "causal trends" here? Suggest shortening this sentence starting at line 72 by cutting out superfluous words. Same with the sentence that follows. ("Among the most visible of these are" is not necessary. I suggest something like "Growing reports on corals spawning during daylight or twilight conditions challenge the understanding in the role of illumination cycles"

- **Authors Response**: We appreciate the reviewer's suggestions, which we have followed in both relevant sentences. We have replaced "causal trends" with "conventionally accepted spawning periods".

Line 77 | Missing a comma in authority (Forskål, 1775)

- **Authors Response**: Thank you, we have made this edit.

Line 80 | "have been explored"

- **Authors Response**: Thank you, we have made this edit.

Lines 80-82 | While it is important to mention the ongoing taxonomic changes, these lines seem to be out of place within this paragraph. I suggest moving the following lines (lines 82 and onwards about other daytime spawner examples) up, right after the *Porites rus* example in lines 77-79. And then end with something like "however these limited observed patterns may be challenged due to ongoing taxonomic changes".

- **Authors Response**: We have broadly followed the reviewer's suggestion by moving the offending lines (former lines 78-82) to the end of the paragraph, allowing for improved

readability and flow. We have retained the wording based on the taxonomic concerns, especially given the recent description of *Pavona giannii* Benzoni, 2025.

Line 89 | The first sentence and the rest of the paragraph do not go together. I understood that published spawning record in general from the Gulf is limited, and this should be mentioned. But the rest of the paragraph is talking about the invasive survey method. The first part (and the very last sentence on line 105) should be a standalone paragraph giving a brief summary of the Gulf of Thailand region and any nighttime and daytime spawning patterns found (or lack thereof) in previous literature. Then a separate paragraph on invasive survey methods and why this is not ideal for already destroyed reefs.

- **Authors Response**: We have followed the reviewer's suggestion and moved the concluding sentences and citations that specifically talk about spawning observations to the follow the start of this paragraph, with a natural lead into the gamete-development assessments and the relevant survey methods.

Line 104 | What is "Appendix A"? I couldn't find any besides Table S1 and two supplementary figures in the document. If it is the figshare video, I believe it should be added in the reference list.

- **Authors Response**: To clarify to the reader, we have replaced the punctuation within the citation to specify that the citation refers to not only the relevant reference, but Appendix A of that reference which focuses on coral spawning.

Lines 109-110 | After reading the Materials and Methods section, I am still unsure what you mean by "only predictive and in situ observational techniques". How are they different from one another with regards to this manuscript? It appears it is better suited to just say "we aimed to identify spawning periods of Pavona for this region"

Authors Response: We have added to this sentence to specify that the methodologies applied are in contrast to conventional techniques used historically (as emphasised in the Introduction), which involve invasive and often damaging techniques over prolonged periods. Predictive and observational, as clarified in the Introduction, are within the criteria of 'non-invasive' methodologies used, in conjunction, with data review allowing for spawning prediction and validated by observation. An example of invasive methodologies are monthly or weekly sampling of coral colonies to examine maturation of gamete bundles, to calculate spawning period, which may or may not be validated by in-situ or ex-situ observation.

Materials and Methods

Line 119 | Unnecessary period after Gulf of Thailand

- **Authors Response**: Thank you we have made this edit.

Line 120 | "This was supported by observations" is this citable? If it was personal communication, please state so

- **Authors Response**: We cited our Table S1 where DMCR records with sufficient data were included in the dataset. We have also clarified the anecdotal and unused data as personal communication with the DMCR.

Line 138 | Were colonies identified to genus or species-level on the spot via live footage, or identified later while viewing recordings? Please clarify

- **Authors Response**: We have added the following sentence to clarify this: "Spawning corals observed via ROV were initially identified to genus and tabulated during the survey, and then were later verified or disregarded based on review of the footage, with the exception of a single survey were footage recording was corrupted."

Line 139 | Very concerned about this medically relevant incident but I think you can just say "growing observations of the *Chironex* sp. box jellyfish (S1 Fig)".

- **Authors Response**: We appreciate the reviewer's suggestion to remove mention of medically relevant incident, however, we do also want to emphasise our numerous observations and justify our concern on this highly understudied issue from the region. We have therefore replaced the end of the sentence as follows: "... due to growing observations and incidents of *Chironex* sp. box jellyfish..." and added relevant references to support.

Lines 146-147 | Is there a list of taxa included in each "null data" point? Table S1 suggests that each observation time point for each location is consolidated into one "null data", however, is it "null" because the taxon (e.g. *Pavona explanulata*) was not found in a given location, or a colony was found but it was not spawning? If there is a list of taxa for each time point at a given location, Table S1 should reflect this as it is valuable information.

Lines 147-149 | What was the result of only giving small credence to this "external" dataset? This sentence is a little unclear so either remove, or give a brief example of how the data was treated. e.g. "If the only observation from the site was from an external observer, the spawning period of the given site was treated as inconclusive"

- **Authors Response** (Lines 146-149): We appreciate the reviewer's concerns on this point which we have addressed and explained as such: We have replaced the suggested sentence (147-149) with a clearer explanation of what criteria were used as null data. These being reef surveys that explicitly observed multiple coral colonies, typically being assessed for their health and ecological factors, but for which no sexual reproductive activity being observed. We have, as suggested, removed the sentence referring to the credence of these observation as we entirely agree with the reviewer that it adds unnecessary ambiguity to the surveys themselves. This has been replaced by a more elaborate clarification of the terminology used. The validity of this methodology is supported by the later inclusion of incidental spawning observations by citizen scientists, and indeed multiple incidental spawning observations made by the authors in this study. Given that the null data surveys were non-specific (looking across multiple genera and species), Table S1 has not been amended to specify any corals for which spawning was not observed. We have instead made reference to our recent work that provides baseline coral community assessments for the majority of sites surveyed, including all null-data survey sites.

Lines 158-159 | "according to World Register of Marine Species (WoRMS)" instead of "according to the WoRMS standard"

- **Authors Response**: We have made the reviewer's suggested edit.

Line 163 | Remove "established"

- **Authors Response**: We have made this edit.

Line 169 | "broader survey regime" does this refer to something specific that was mentioned before? Please clarify

- **Authors Response**: We thank the reviewer for pointing this out, it was an accidental remnant of an earlier draft of the manuscript, which has now been removed.

Lines 174-189 | Under the heading Data analysis, there are a fair bit of parentheses which I suggest removing for readability. For example, sentence starting at line 177 can be turned into a semicolon-separated list starting with "For all data including spawning observations from present study and previous studies compiled, and null data, the following information was collected where relevant: A; B; and C." Another example: the parentheses for line 187 syzygy is unnecessary as the term has been defined in lines 185-186.

- **Authors Response**: We appreciate the reviewer's suggestions in this section which we agree improve the readability. We have made the suggested edits accordingly.

Results

Throughout | If using a 24-hour clock, "pm" is unnecessary. Please check throughout the manuscript (including in Discussion where a 12-hour clock was used).

- **Authors Response**: We have made the suggested edits throughout the manuscript, with the exception of a single case in the discussion where we make direct reference to the time stated by Lin and Nozawa (2017) and therefore prefer to quote verbatim.

Figure 1 and throughout | Please use higher resolution figures, as currently for figure 1, I could barely make out the species names and the black section of the pie chart that indicates spawning duration (e.g. I cannot see a black pie for Koh Larn, *Dipsastraea* sp. in Hin Ploeng, Koh Rangka Jeaw, *Pavona explanulata* in Sail Rock etc., but please see comments below on Figure 1 specifically).

- **Authors Response**: The reviewer's comment is somewhat concerning as all figures submitted to the journal are already at very high resolution (min. 3000px; >1mb for vector graphics). Below is a screenshot of a zoomed-in section of Fig. 1 for reference. We believe that the loss of resolution of images available to the reviewer are likely due to excessive compression by the journal's submission platform. Therefore, in an effort to allow the reviewer to properly assess the resolution of our figures, we have included them, with captions, at the end of our responses to reviewers.

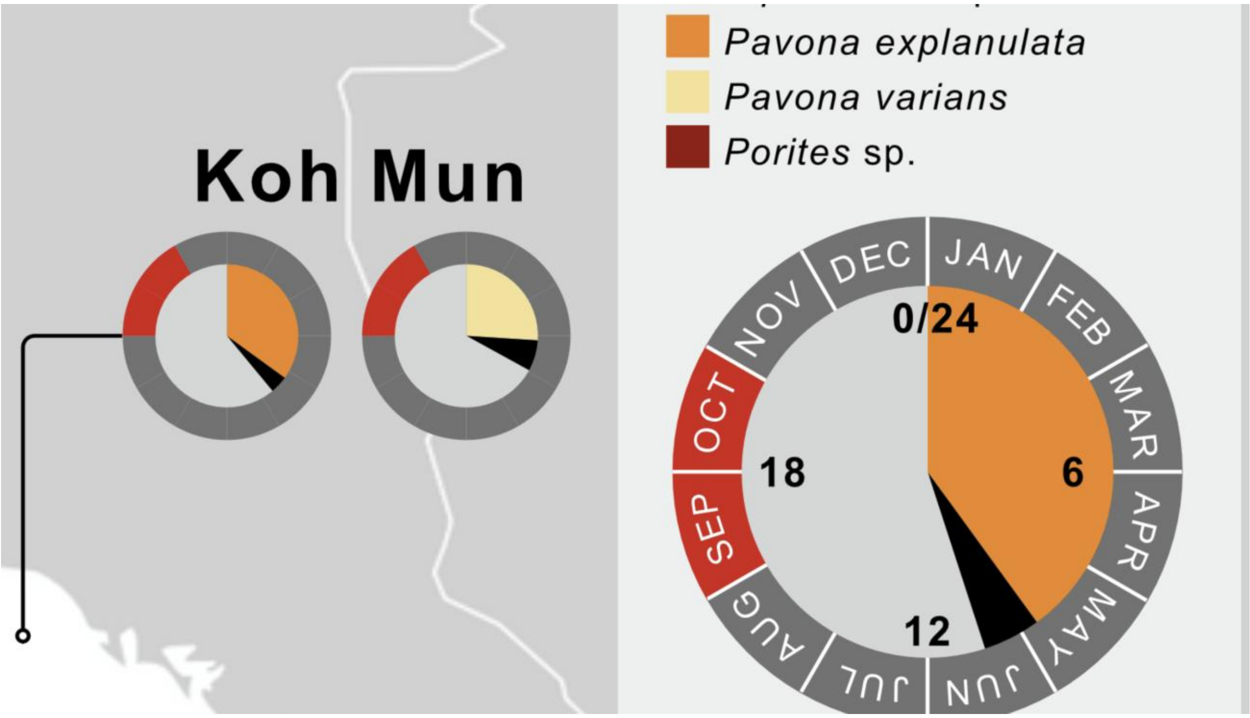

Line 198 | Change from (Fig. 2) to (Fig. 2G)

- **Authors Response**: Thank you, we have made this edit.

Line 200 | Table S1 observation No.49 suggests that *P. explanulata* started spawning at 13:12 in Koh Koun, Cambodia. Please clarify.

- **Authors Response**: We thank the reviewer for pointing this out, this was an incorrect time in the results section and we have replaced this with the correct earliest time as 13:12pm. Additionally we have specified the single period of spawning overlap between the two species during a given spawning at a given location being 7 minutes on the 11th of October 2022.

Lines 214-216 | It is not clear whether the whole sentence is referring to the black portion of the pie only, or the other coloured pies as well. I suggest starting the sentence with "Black pie indicates…" if the sentence is only referring to the black portion of the pie.

- **Authors Response**: We have made the reviewer's suggested edit and significantly improved the caption to add clarity (see below).

Figure 1 | (1) Relating to the comment on Lines 214-216 above, it is not clear what the coloured piece of the pie chart is indicating besides what species it is representing. Currently it could be interpreted as "time since sunrise to onset of spawning", however this should be unnecessary if the black pie is shown. I suggest either colouring in the whole circle to indicate species or change to a simple text label for each species. (2) Is there a reason the black pie is not shown for some locations/species? If so, please clarify in the figure caption. Looking at Table S1, it seems that in some cases the black pie is not shown in Figure 1 even there were spawning start and end times recorded because there was only one observation? (e.g. Koh Larn, *Pavona varians*) If there was a minimum threshold to make it into the pie charts, please clarify in the figure caption.

- **Authors Response**: We thank the reviewer for not only assessing the figures, but also stating their interpretation of the figure better allowing us to improve the caption to add appropriate clarity. Therefore, we have amended the wording captions of Fig. 1 and Fig. 6, including an added explanation of the example provided in the legend, to clarify. First and foremost, we have removed the word ‘’pie’’ from captions and descriptions which undoubtedly contributes to misinterpretation of the charts. To address the reviewer’s concern here directly, the coloured section of the chart does indeed serve to identify the species in question, while simultaneously indicating the earliest recorded **start** of spawning among the recorded days. A major point of our paper is the quantity of hours after sunrise (aka daylight) before the start of spawning, and that we hypothesise a possible daylight cue. Therefore, believe our clarification in the caption and text reduces the opportunity for misinterpretation. Our rewording targets the misinterpretation that these simply show the start and end of spawning, instead emphasising quantity of hours post sunrise, and visualising the natural variation recorded across the data for the earliest start of spawning timing alone. The black section of the chart indicates the latest **start** of spawning for that taxon at that location among the recorded days. We have also specified that pie charts without black sections indicate only a single spawning record or nearly identical earliest spawning record. As for the reviewer’s recommendation to replace the colours with added labels, alongside the added labels for provinces and borders, we find that level of visual complexity in the figure drastically increases as a result. We maintain that the current chart format remains the clearest way can envisage to combine scale, variation and detail in temporal data of spawning, combined with taxonomic and location detail. We have attempted with each figure to keep data visualisation to be uncluttered while informative and have therefore chosen to greatly elaborate the figure captions rather than making excessive edits to the data visualisation.

(3) My apologies as I am not familiar with the location names, but I cannot see data from "Koh Rangka Jeaw" in S1 Table. Is data for this location under a different name? Also I see that there are a fair few *Pavona* daytime observations from "Hin Yuan" in S1 Table but they are not apparent on Figure 1. Please clarify why some locations were chosen to be shown over others.

- **Authors Response**: The need for improved location labelling has been noted and we have added a column to table S1 to add further spatial resolution by distinguishing individual surveyed sites in comparison to larger islands/island groups that spatially distinct. We have also added the province labels and delineations to the figure to further help with cross-referencing as per reviewer's suggestion. We also appreciate the reviewer identifying that Koh Rangka Jeaw was not represented in our supplementary data. Upon review, this was an error on our part due to the aforementioned need to establish multiple local references for location information. Line 493 has been corrected from it's previous ''Chumphon Marine Park'' to the more specific ''Koh Rangka Jeaw'', in Chumphon province.

Lines 221-222 | What does the red square in Fig. 2B indicate? Is this where Fig. 2A was cropped out of? Please clarify in the figure caption. I am assuming this square was added onto the image later. If not, please ignore this comment.

- **Authors Response**: Thank you for this suggestion, we have corrected our caption to clarify the purpose of the red box as indeed a zoomed-in crop to more easily show granularity of egg release.

Line 234 | Does this line correspond to Fig. 2C image? If so, please add (Fig. 2C) after "... confused for sperm release".

- **Authors Response**: We have made the necessary edits and specified reference to Fig. 2A, thank you.

Line 246 | Same as Line 234, is this explaining the Fig. 2C image? Also suggest choosing either translucent or transparent

- **Authors Response**: We have made the necessary edits and specified reference to Fig. 2C as suggested, thank you.

Line 247 | Suggest "...all other daytime spawning non-agariciid corals" and remove the parentheses.

- **Authors Response**: Thank you, we have made this edit.

LInes 253-254 | Suggest spelling out the rest of the *Platygyra* trend, e.g. spawned between 102 to 160 hours after lunar peak

- **Authors Response**: Thank you for the suggestion, we have made this addition.

Lines 254-256 | Suggest adding the conversion to days in the text or changing the scale in Figure 4 to hours instead of days for consistency

- **Authors Response**: Thank you for the suggestion, we have made these edits to the text.

Lines 256-260 & Figures 1&4 | (1) Would it be possible to add the province labels to the map in Figure 1? Reading the lines 256-260 and looking at Figure 4, it is not immediately clear where the boundaries are between provinces and islands, and it will help those of us not from the region.

- **Authors Response**: Thank you for this suggestion, we agree that this simple edit can greatly improve comprehension for those unfamiliar with Thai provinces, we have made this update and edited the text to clarify these.

(2) It seems in Figs. 4C and 4D, the blue dots for Chumphon are at least 30 days apart from the rest or quite a difference between -15 days vs ~-2 days from full moon. Unless there is another dot inside the cloud that I cannot see, I believe this qualifies as a distinct pattern. So I suggest either adding "... however, differences were observed between eastern and western Gulf" after the "No discernible differentiation was observed..." sentence, OR change the first sentence to "No consistent geographic pattern was observed ... except *Pavona* in Chumphon region spawned a month apart from the rest".

- **Authors Response**: The reviewer's observation that the Chumphon observation is distinct from the others is apt and therefore we have dedicated much of the text in indicating this. However, the entirety of the Chumphon dataset is based on a single (days) observation by Plathong et al. (2006), and is not the only observation from the western Gulf. One colony from Sail Rock (Surat Thani) of *P. explanulata* (from Monchanin et al., 2021) fits comfortably with the remaining observations of the species. Therefore, while we agree that the observation is distinct, we disagree that this qualifies as a pattern, as it is based on a single observation, with no indication to suggest null data on either side of the observation was carried out. Therefore, we ascribe similar credence to the Chumphon observation being indicative of a valid and distinct ''trend'' as we do it being an entirely anomalous observation that may be caused by a wide range of unknown factors. Given the extent to which this distinction is discussed in the text, and our preference to keep our Results section pertinent to data with greater support (multiple surveys, greater temporal resolution, etc.), we have not made the reviewer's suggested edit in this instance.

Figure 4 | Suggest making the text sizes on the axis titles and labels larger as there seems to be enough space in the plots.

- **Authors Response**: Thank you, we have made this edit for all labels in Fig. 4.

Figure 6 | Similar to comments for Figure 1, the coloured pie is misleading as is, if I am understanding this correctly. The coloured pies currently can be interpreted as spawning has been observed since 0 hours after sunrise for every species and location, as opposed to what you have reported in lines 280-283 "at least 12 hours after sunrise". If using this figure format, each coloured pie should start where the earliest spawning time has been recorded, not at the top of the clock at 0 hours since sunrise. And according to the lines 292-295 figure caption, because Figure 6 is only showing the onset of spawning and not the whole duration of spawning, I believe each circle shouldn't be a pie, and instead should be a single line (or a thinner black pie like in Figure 1 to show variation in start times).

- **Authors Response**: As per our response to Fig. 1, we have reworded this caption to emphasise that this chart highlights the quantity of time after sunrise before the start of spawning. We have made this explicit by proving another example of the legend provided. While the reviewer's initial misinterpretation is understandable, our goals of visualising multiple distinct variables (time, month, species and location), and emphasising the quantity of time from sunrise, are achieved by the current format.

Additionally, for most locations, multiple spawning start periods are not provided, and thus we have not felt it useful to retain the black sections as in Fig. 1. We are hopeful that our rewording and clarification prevents further misinterpretation of these charts indicating start and end of spawning, but instead the quantity of daylight hours prior to the start of spawning.

Discussion

Lines 300-303 | Although an important aspect, I believe this sentence takes away from the key finding of this paper, which I understood to be the overall coral spawning pattern in the Gulf with particular focus on two daytime spawning *Pavona* species. I believe this section under the heading "Synchrony in the Gulf of Thailand" should be separated into two, and thus, reorganise text accordingly: one, to describe in detail the spawning synchrony observed among species and comparison with previous literature from the region; and another under a heading such as "Non-invasive methods to assess spawning synchrony" to discuss pros and cons of the method.

- **Authors Response**: We appreciate the reviewer's suggestion and agree that splitting these perspectives improves the overall comprehension. As such, we have moved the first sentence to the start of the new section "Assessing Non-Invasive Methods to Study Coral Reproduction" below the section discussing synchrony. Our wording for this section differs slightly from the reviewer's suggestion as we have noted that histological and other more invasive methods have been historically used to assess more than just spawning. We have also reorganized this part of the discussion to more cohesively adhere to the separated sections accordingly.

Lines 317-320 | See comment in the Results section lines 256-260. I believe the lines here should be in the Results section, then further discussed in the Discussion (like you have in lines 320 and onwards).

- **Authors Response**: We have moved the indicated lines to the results as suggested and edited the start of this paragraph to more explicitly discuss the results rather than presenting them.

Line 329 | ...was found "to" differ by

- **Authors Response**: Thank you, we have made this edit.

Lines 334-342 | I understood that non-invasive method has limitations, but I am not sure I understand what the rest of the paragraph means. Are you suggesting that based on previous species abundance data, there should be more spawning information on the most abundant taxa e.g. *Astreopora*, *Leptastrea*, *Pavona*, but there isn't any in the literature? And that this present study also did not observe any spawning for those abundant taxa? If this is the case, I suggest spelling that out; for example, "Based on previous biodiversity surveys, we had expected to observe spawning of the two most abundant groups *Leptastrea* and foliose *Pavona*. However, throughout the 5-year survey in present study no spawning was observed for these groups". Additionally, if the focus here is only Agariciidae, perhaps remove *Astreopora* from the list of genera.

- **Authors Response**: We appreciate the reviewer's suggestions regarding this section. As a result, we have reorganized and rewritten most aspects to improve comprehension and more explicitly state the point we are trying to make.

Line 357 | Suggest a heading that is a little more descriptive. Since this section only talks about *Pavona* corals, I suggest something along the lines of "Global spawning patterns of *Pavona*"

- **Authors Response**: Thank you, we appreciate the reviewer's valid suggestion and we have edited accordingly.

Lines 372-381 | (1) Parts of this paragraph should be in the Results section, as the only text about the regional differences in spawning time within the year I found was at line 275 "...yet latest in the year", and line 281 "... spawning across various times of the year".

- **Authors Response**: The reviewer's concerns are noted, and we concede the need for some further elaboration regarding this topic in the Results section. We have therefore moved parts of the relevant section to the Results to further elaborate on the regional differences. However, we have left much of this section in place (with many modifications) as it is largely still a discussion and exploration of findings presented, contextualised by historical and geographic data. With the elaboration of relevant aspects in the results section, we hope that it is now more relevant and clearer to the reader the need for these topics to be discussed in this section.

(2) I suggest breaking this part into two parts for readability: one discussing the annual pattern differences (January-May & June spawning in Panama & Hawaii vs September-December spawning in Gulf of Thailand vs Red Sea); and two the differences or lack

thereof within the lunar cycle (spawning concentrated around fullmoon to 4 days after). Also I'm not sure what you mean in the parentheses "(with all records between N22° and N7°)", please clarify.

- **Authors Response**: We have reorganized the sentences to divide the annual and lunar discussion points more clearly. Additionally, we have removed the section in the parentheses identified to avoid confusion. While our intention was to demonstrate the geographical constraint of observations, the point in general adds little value while raising only ambiguous questions.

(3) In line 379 "more heavily influenced", this implies that this idea was exclusively tested e.g. with synchrony index (see Bouwmeester et al. 2021 Coral Reefs). Perhaps the wording can be changed to highlight that the observed pattern **coincided** with longitudinal separations. However, for exploratory purposes I suggest you at least mention other possible factors such as temperature regimes and taxonomic uncertainties just like in the introduction lines 58-59. I can see that light regimes and taxonomic uncertainties are discussed more later under "Broader Insights", so perhaps only list possible factors here, then write "see below".

- **Authors Response**: We appreciated the reviewer's suggestion, in particular identifying indeed that we mistakenly suggest an influence (ye olde trap of correlation is not causation). We have followed the reviewer's suggestion in this rewording and have added a suggestion mentioning other possible factors.

LIne 384 | Was this meant to be a citation (in [number] format) or a typo for "from Taiwan by Lin and Nozawa [57]"?

- **Authors Response**: Thank you for catching this, this was missed in our formatting!

Line 393 | Remove apostrophe from "author's"

- **Authors Response**: Thank you, we have made this edit.

Line 408 | Possible typo of "recorded as"?

- **Authors Response**: Thank you, we have made this edit.

Line 409 | Change "spawning after sunset " to "spawn after sunset"

- **Authors Response**: Thank you, we have made this edit.

Line 412-413 | (1) I believe these *Pavona* spp. are the ones referred to as the foliose *Pavona* at lines 334-342. This information "(abundant across the Gulf of Thailand)" should be presented earlier if this is the case.

- **Authors Response**: Thank you, we appreciate the reviewer's valid suggestion, we have expanded on this point based on our recent work at the suggested section (formerly lines 334-342), however we have maintained mention of abundance in this specific line to re-emphasise the point.

(2) Was *P. frondifera* recorded in this present study? I can only see *P. decussata* in Figure 5B. If the claim of *P. frondifera* being a nighttime spawner is based on null data, that distinction is necessary.

- **Authors Response**: Thank you, we appreciate the reviewer's valid suggestion and we have edited accordingly.

Line 414 | Suggest "Across the genus-level" instead of "When looked at in aggregate"

- **Authors Response**: Thank you, we appreciate the reviewer's valid suggestion and we have edited accordingly.

Lines 415-417 | Suggest breaking this sentence into at least two for readability.

- **Authors Response**: Thank you, we appreciate the reviewer's valid suggestion. We have reviewed this section and added some breaking to sentences and sentence structure.

Line 417 | "correlated"

- **Authors Response**: Thank you, we have made this edit.

Line 424 | Suggest a more descriptive heading. Perhaps add "into daytime spawning".

- **Authors Response**: Thank you, we appreciate the reviewer's valid suggestion and we have edited accordingly.

Lines 431-432 | Which description? In the methods section lines 156-158, citations indicate that series of recent literature was used, not the original description by Verrill 1864.

- **Authors Response**: We have clarified in this section that the original descriptions (Lamarck's in French and Verrill's in English) were reviewed, as well as more comprehensive material in recent years. We have also added this to the relevant section in the methodology to avoid confusion.

Lines 429-436 | I believe there are two ideas presented here. One, that your data from Gulf of Thailand identified *Pavona* species using multiple lines of evidence, so adding confidence that each *Pavona* species are good distinct species. The second idea is that the global dataset of *Pavona,* for example the putatively widely distributed *P. varians* may contain multiple species. This is also valid because three nominal species are currently junior synonyms of *P. varians* (Verrill, 1864): *P. repens* (Brüggemann, 1877), *P. intermedia* (Gardiner, 1898), and *P. percarinata* (Ridley, 1883). If you look at the original description of each species, you can see that they are all described from different locations. And like it says at the end of the paragraph (lines 435-436), molecular techniques may also reveal that there are other undescribed species, so it is possible that the regional differences are due to "*P. varians*" containing multiple species. In short, I suggest reorganising this paragraph so that it highlights that (1) the global dataset of one "widely distributed species" may contain multiple species, and (2) each of your study taxon is identified as distinct species unit based on multiple lines of evidence, so it is a valid dataset no matter what the identity of the organisms may be.

Having said that, if you do reorganise this paragraph this way, I suggest including images of the representatives of key species as supplementary material (just like Figure 2). That way there is a better chance of future proofing your data in case of taxonomic updates.

- **Authors Response**: The reviewer makes a valid and interesting point. We had refrained from delving too deep into the taxonomy of the group given the scope of this work, however elaboration does support the discussion (especially given the recent description of *P. giannii*). We have therefore split this paragraph in two as suggested, with added sections elaborating on the taxonomic points. Additionally, we have created a new supplementary figure (Fig. S3) as suggested to provide some additional visual material regarding the two putative species identified in our spawning dataset. We have nonetheless attempted to keep this aspect of the work concise to allow for appropriate length of other, more directly related parts of the discussion.

Lines 442-444 | I expected to see a longer list of citations with a sentence starting "There is no shortage of literature". Suggest adding relevant literature that is already in the reference list (such as the same ones from the Introduction). Also suggest "nighttime" spawning time here.

- **Authors Response**: We appreciate the reviewer pointing out this oversight, we have made the recommended edits and additions.

Lines 468-469 | Perhaps add "earlier" before daytime spawning? Other Pavonas from the global dataset also spawns during daytime.

- **Authors Response**: Thank you, we appreciate the reviewer's valid suggestion and we have edited accordingly.

Paragraph between lines 463-488 | It seems that there are several ideas presented in this paragraph which makes it difficult to comprehend. The opening sentence suggests that photoprotective mechanisms may differ between nighttime and daytime spawners, but the following sentences do not compare known mechanism in typical nighttime spawners vs daytime spawners. I suggest re-writing this paragraph for clarity.

- **Authors Response**: We have somewhat addressed this concern by creating a new paragraph to further elaborate on the sparse literature exploring the impact of UV radiation on coral larvae. We have suggested that there may be differences in day and night-time spawning corals with regards to photoprotection (given solar insolation), but this question has never been explored as records of day-time spawning are as sparse as the aforementioned literature. This paragraph is followed by the separate section exploring *Pavona*-specific and Thailand-specific observations and inferences, allowing for a more clear distinction in discussion points.

Lines 489-493 | This should go in the introduction, around lines 74-78. Then the sentence can simply start with "Our data contributes to the growing observation of daytime spawning in scleractinian corals. In particular, ..."

- **Authors Response**: Thank you, we appreciate the reviewer's valid suggestion, we have the specified sentence to the introduction as suggested. However, this has been included in the culiminating paragraph of the introduction as opposed to lines 74-78, to maintain a cohesive structure to the section.

Lines 506-508 | This sentence contain 43 words. I would remove unnecessary words such as "certainly", and separate this into at least two sentences e.g. "Further investigation is needed for the geographical extent of daytime *Pavona* spawning. Questions also arise whether fertilisation and recruitment remain viable under UV exposure ..."

- **Authors Response**: Thank you, we appreciate the reviewer's valid suggestion and we have edited accordingly.

Line 515 | due "to"

- **Authors Response**: Thank you, we have made this edit.

Lines 529-534 | This seems out of place and while interesting, rather irrelevant to the manuscript. Perhaps keep it in the supplementary or a separate observation note?

- **Authors Response**: Thank you, we appreciate the reviewer's valid suggestion, have removed this section.

Reviewer #3:

You document clear, daytime, synchronous spawning windows for Pavona explanulata and P. varians in the eastern Gulf of Thailand/Cambodia and add a useful global recap for Pavona. The findings are relevant and actionable. Most of my notes are about how the figures and wording could be tighter.

1. Fig. 1&6. Right now the plot shades from 0 h (sunrise) all the way to the "latest onset," which reads like "spawning might start anytime from 0–X h." If the real window is 6–8 h after sunrise, only shade 6–8 h. Add a tick for the median and a band for IQR/95% CI if it is available.

- **Authors Response**: We have amended the wording captions of Fig. 1 and Fig. 6, including an added explanation of the example provided in the legend, to clarify. Specifically we aim to show not the period of spawning, but the period of daylight prior to the onset, with the quantification therefore not being spawning period, but rather daylight period prior to spawning. A major point of our paper is the quantity of hours after sunrise (aka daylight) before the start of spawning, and that we hypothesise a possible daylight cue. Therefore, believe our clarification in the caption and text reduces the opportunity for misinterpretation. Our rewording targets the misinterpretation that these simply show the start and end of spawning, instead emphasising quantity of hours post sunrise, and visualising the natural variation recorded across the data for the earliest start of spawning timing alone.

  2. Line 49-51. The current sentence implies both modes disperse "gametes" (eggs + sperm) by currents. That fits broadcast spawners but not brooders (who retain eggs, release sperm, fertilize internally, then release larvae).
- **Authors Response**: We appreciate the reviewer's comment, we have reworded these sentences to clarify these points. However, while it is true that brooding corals disperse over shorter distances, it is absolutely also true that hydrodynamic factors play a major role in both gamete dispersal as well as influencing larvae/planula prior to settlement. If sperm release happens during a strong eastward current, there are no mechanisms the authors are able to find that would allow a westward fertilisation of brooding colonies, for example.

3. Most Dipsastraea are hermaphroditic broadcast spawners with nocturnal bundle release. Daytime sperm-only is atypical and can be confounded by upstream sperm plumes or viewing angle.

Suggestion: label as “Sperm (putative)”, describe your scoring criteria (continuous release from the focal colony, no bundles observed, clear source), and discuss it conservatively.

- **Authors Response**: We have followed the reviewer’s suggestion and added a sentence in the Results accordingly.

Copies of updated and uncompressed figures for more appropriate reviewing:

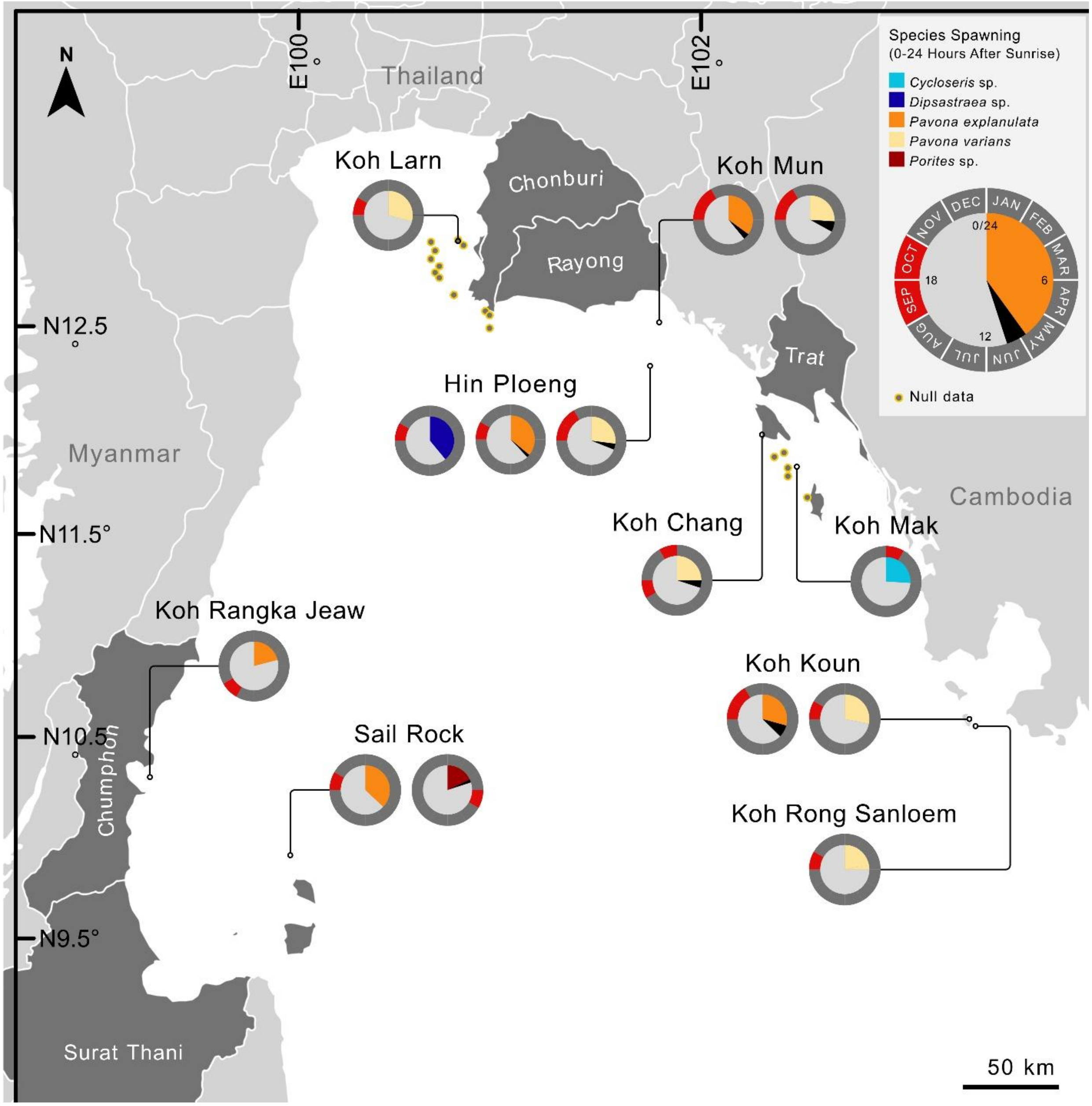

Fig. 1 Daytime spawning corals across the Gulf of Thailand. Dataset includes present surveys and previous records from the Western Gulf of Thailand. Coloured section of charts indicate briefest quantity of minutes and hours after sunrise (out of a total 24) at the start of spawning, with latest recorded start of spawning for each coral and location indicated in black. Outer ring of the pie chart indicates the total months of the year during which spawning was identified per species per location. Sites which were surveyed but where no spawning was recorded are identified as null data. Legend provides an example of *Pavona explanulata* indicating earliest start of spawning period to be approx. 9 hours and 50 minutes after sunrise, with the latest start of spawning being approx. 10 hours and 50 minutes after sunrise. Legend indicates all spawning records to be September and October. Pie charts without black sections indicate only a single spawning date recorded or identical spawning onsets. Detailed timings accessible in Table S1. The basemap incorporates provincial boundary data from SimpleMaps (CC BY 4.0).

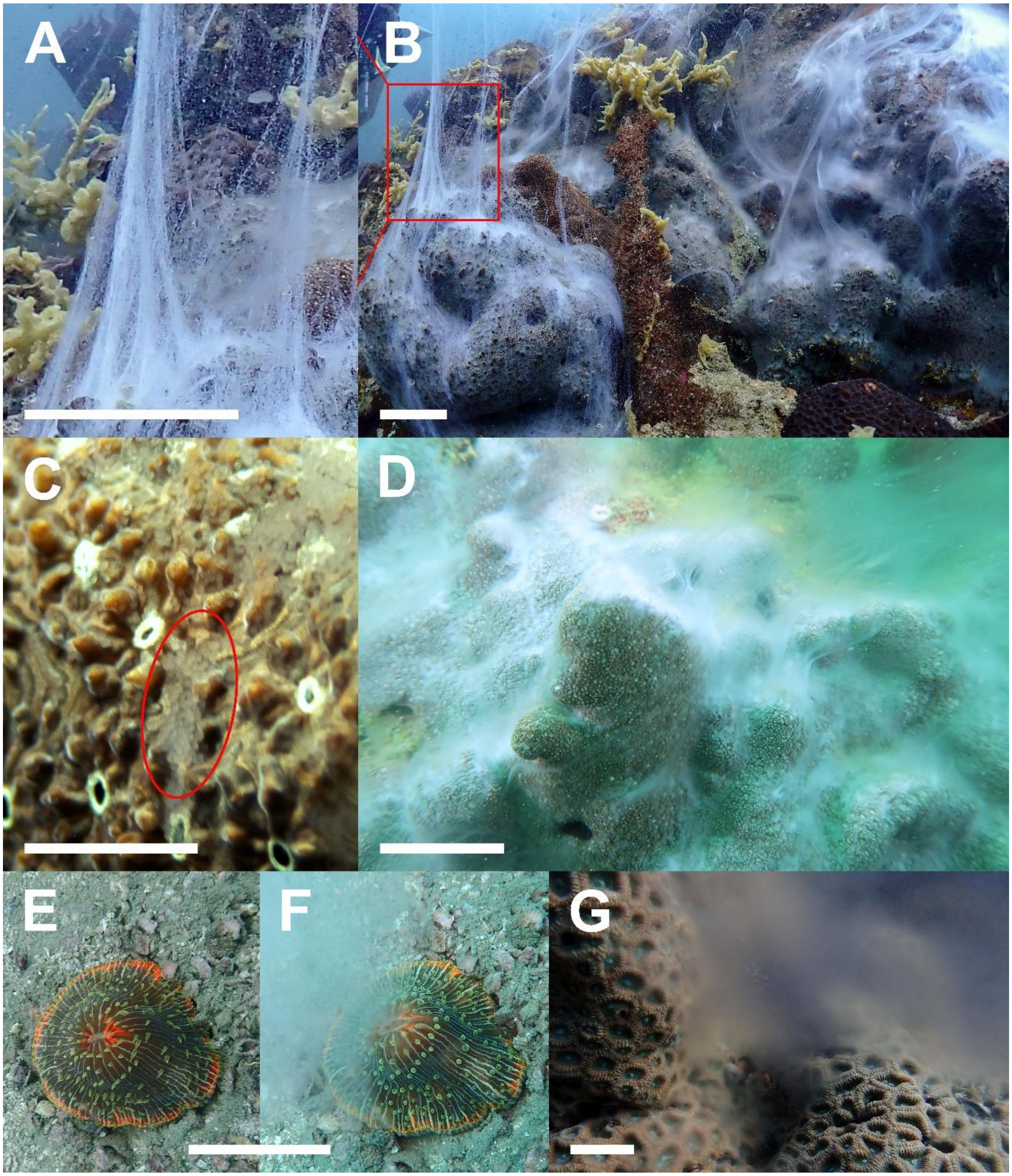

Fig. 2 Photographs of daytime gamete release of corals recorded along the Eastern Gulf of Thailand. A) Egg release by partial colony of *Pavona varians* (scale bar 50mm); B) Simultaneous egg and sperm release by a hermaphroditic colony of *P. varians* (scale bar 50mm), red box indicates portion zoomed in for (A) to more easily differentiate egg release from sperm; C) Near-transparent egg-release (red ellipse for example) by polyps of *P. explanulata* (scale bar 10mm; photo by Thomas Thana Real); D) Whole-colony sperm release by *P. explanulata* (scale bar 50mm); E) and F) Photos taken five seconds apart before and during gamete release by *Cycloseris* sp. (scale bar 50mm); G) Sperm release by *Dipsastraea* sp. (scale bar 50mm).

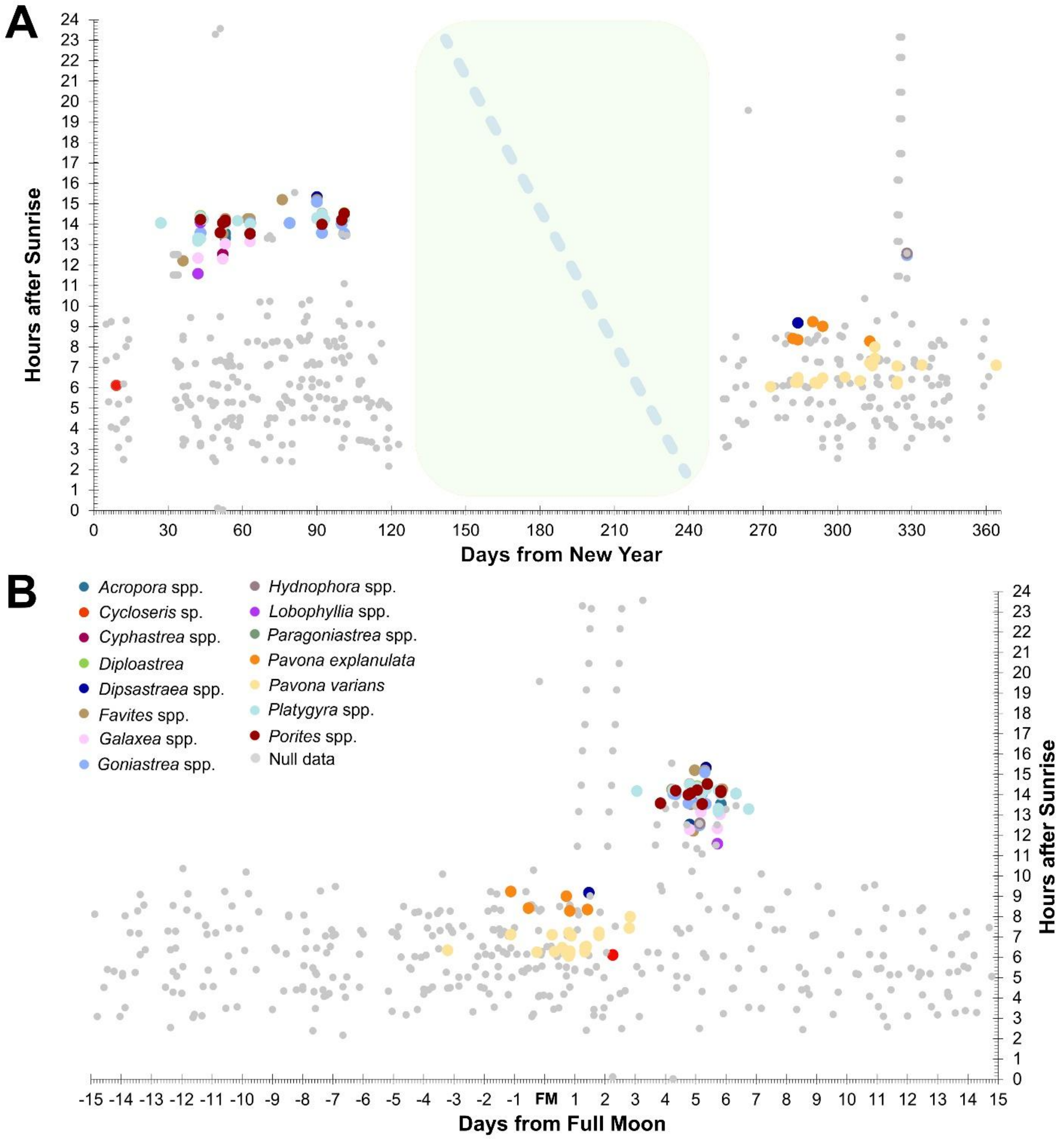

Fig. 3 Plots of spawning timing of all recorded scleractinian corals, and null data, along the Chonburi, Rayong and Trat provinces of the Gulf of Thailand between 2021 and 2024. A) Records of spawning in hours after sunrise plotted against days after new year with monsoon period preventing surveys indicated in the middle of the year; B) Records of spawning in hours after sunrise against days from full moon.

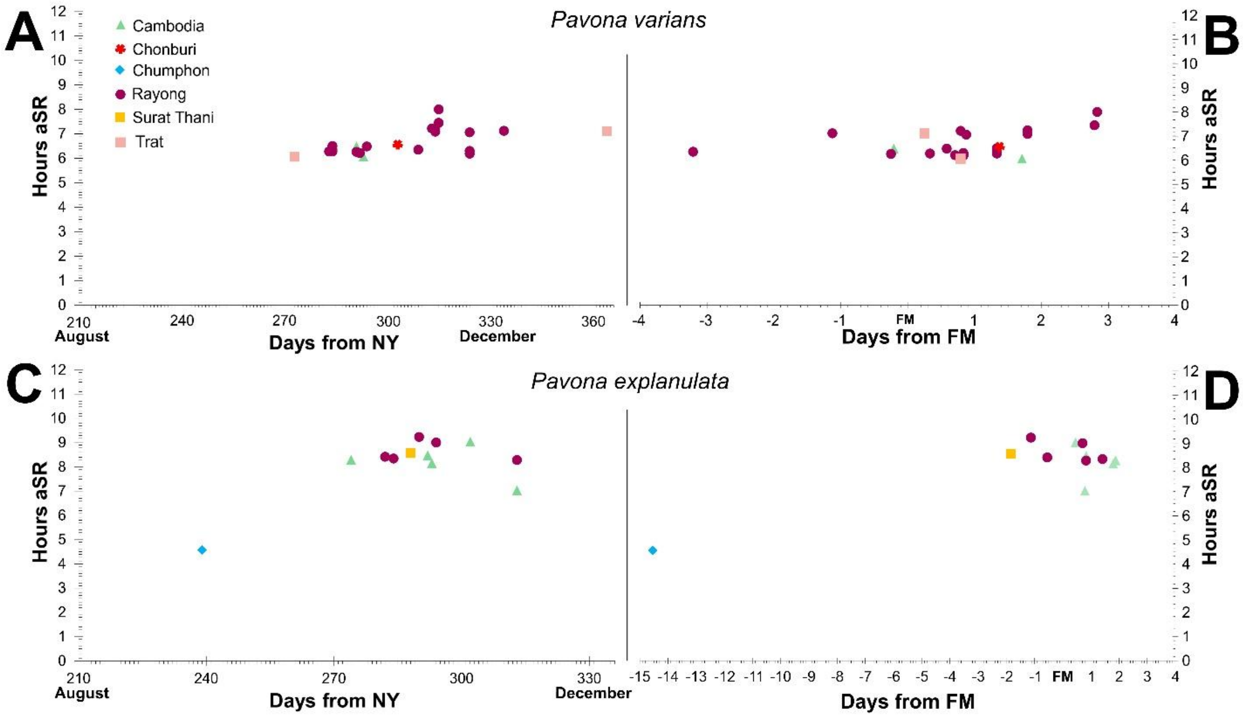

Fig.4 Plots of spawning timing for *Pavona varians* and *P. explanulata* along the Gulf of Thailand (by province) and Cambodia between 2021 and 2024. Spawning timing of *P. varians* with hours after sunrise plotted against (A) days after new year (emphasising the second half of the year) and (B) days after full moon, and *P. explanulata* showing hours after sunrise plotted against (C) days after new year and (D) days after full moon.

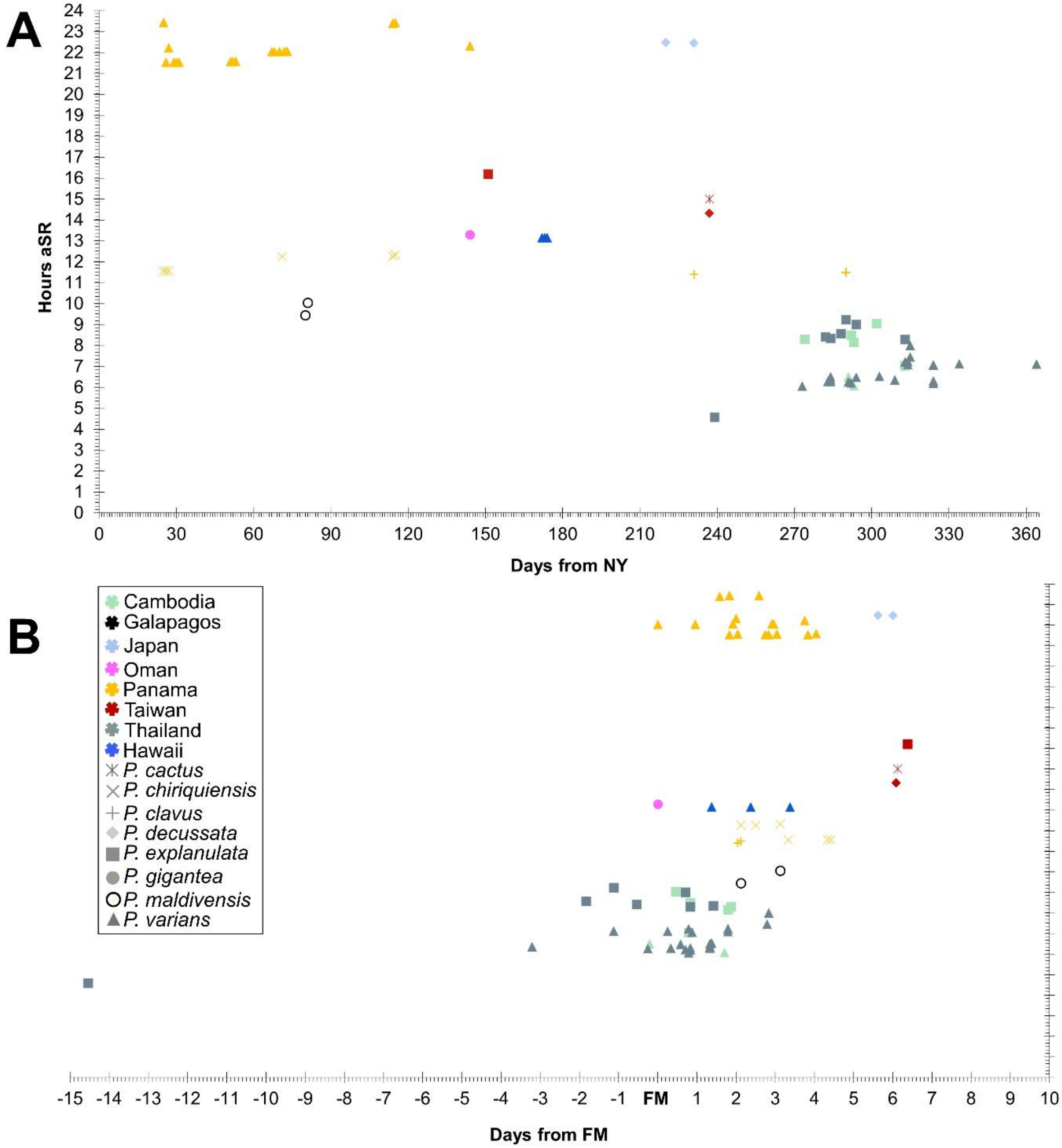

Fig. 5 Plots of spawning timing for *Pavona* corals across the globe by species and location. A) Records of spawning in hours after sunrise plotted against days after new year; B) Records of spawning in hours after sunrise against days from full moon.

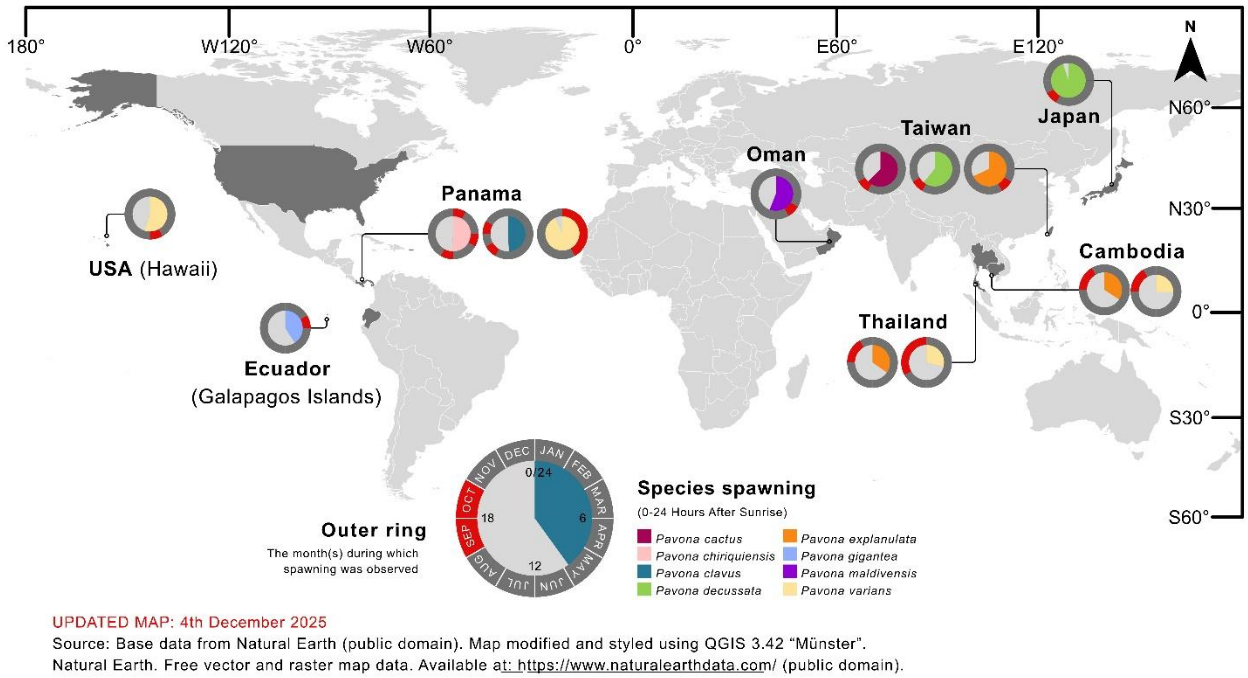

Fig.6 Spawning timing of *Pavona* corals across the globe, by species. Charts indicate briefest quantity of minutes and hours after sunrise (out of a total 24) at the start of spawning. Outer ring of the pie chart indicates the total months of the year during which spawning was identified per species per location. Legend provides an example of *Pavona clavus* indicating earliest start of spawning period to be approx. 9 hours and 50 minutes after sunrise, with all spawning records to be September and October. Detailed timings accessible in Table S1. The basemap incorporates provincial boundary data from SimpleMaps (CC BY 4.0).
