# Supplementary material for: Coral spawning patterns in the Gulf of Thailand reveal synchronised annual daytime spawning, with a review of spawning patterns in Pavona corals across the Indo-Pacific
Source: PLoS One. 2026 Feb 20;21(2):e0343346. doi: 10.1371/journal.pone.0343346 (PMC12922984; doi:10.1371/journal.pone.0343346)
Supplement: Attachment — Submitted filename: Review_PONE-D-25-47186.docx [file pone.0343346.s005.docx]

PLOS ONE Manuscript Number: PONE-D-25-47186

Article Type: Research Article

Full Title: Daytime spawning corals in the Gulf of Thailand and a review of spawning patterns in Pavona corals across the Indo-Pacific

---

**General comments to the authors:**

The manuscript "Daytime spawning corals in the Gulf of Thailand and a review of spawning patterns

in Pavona corals across the Indo-Pacific" offers invaluable information on scleractinian coral spawning in the Gulf of Thailand over multiple years, with specific focus on daytime spawning *Pavona* species. It sheds light on taxa that are often overlooked because they do not spawn during the conventional spawning time that is after sunset. By paying attention to the daytime spawners and employing creative solutions, the authors demonstrate that the overall coral spawning periods in the Gulf are between September to December and February to April. Curation of null data also allowed for further refinement of the spawning periods in this target region. The data comparison between other regions highlights that regional differences exist for month of spawning and time of the day, but not for days relative to full moon. These findings will greatly benefit researchers in coral reproductive biology and restoration projects, and advance the overall knowledge in coral spawning. Below, I list questions and minor suggestions that may help improve the manuscript.

---

**Specific comments and questions to the authors:**

I suggest thoroughly checking for present vs past tense; e.g. line 142 "...are included in our dataset". Also suggest checking for word repetitions or tautology; e.g. line 24, suggest removing "across the globe" as it is repetitive after "Global documentation"; lines 347-348 in Discussion needs rewording "reliable coral spawning ... rely on ...". Check for consistency of a word or phrase used, e.g. "daytime" and "day-time". Some of the sentences are very long (30+ words) and I suggest those to be separated or rewritten.

**Absract**

Lines 35-37 | Suggest working in something like ", contrasting to the February to April spawning periods for nighttime spawners" to highlight the differences between daytime and nighttime spawners.

Lines 38-39 | Perhaps "consistently observed" or "consistent window" is better than saying "highly predictable", because you provide empirical evidence that they indeed spawned, though this may be preference.

**Introduction**

Lines 47-51 | Suggest rewriting these two sentences as they are both currently long and difficult to read. For example, "Scleractinian corals reproduce asexually and sexually. Asexual reproduction occurs due to fragmentation and results in colony formation and growth. Sexual reproduction contributes to population turnover and adaptation through natural selection, and happens either externally (broadcasting) or internally (brooding). Sexual reproduction by both external and internal means rely on the release of sperm and hydrodynamics to disperse gametes across the reef."

Line 55 | This sentence is misleading, as "over 100 species...all spawning in a single night" is not what Babcock et al. 1986 showed. Please clarify.

Line 66 | Remove "obvious"

Line 74 | What do you mean by "causal trends" here? Suggest shortening this sentence starting at line 72 by cutting out superfluous words. Same with the sentence that follows. ("Among the most visible of these are" is not necessary. I suggest something like "Growing reports on corals spawning during daylight or twilight conditions challenge the understanding in the role of illumination cycles"

Line 77 | Missing a comma in authority (Forskål, 1775)

Line 80 | "have been explored"

Lines 80-82 | While it is important to mention the ongoing taxonomic changes, these lines seem to be out of place within this paragraph. I suggest moving the following lines (lines 82 and onwards about other daytime spawner examples) up, right after the *Porites rus* example in lines 77-79. And then end with something like "however these limited observed patterns may be challenged due to ongoing taxonomic changes".

Line 89 | The first sentence and the rest of the paragraph do not go together. I understood that published spawning record in general from the Gulf is limited, and this should be mentioned. But the rest of the paragraph is talking about the invasive survey method. The first part (and the very last sentence on line 105) should be a standalone paragraph giving a brief summary of the Gulf of Thailand region and any nighttime and daytime spawning patterns found (or lack thereof) in previous literature. Then a separate paragraph on invasive survey methods and why this is not ideal for already destroyed reefs.

Line 104 | What is "Appendix A"? I couldn't find any besides Table S1 and two supplementary figures in the document. If it is the figshare video, I believe it should be added in the reference list.

Lines 109-110 | After reading the Materials and Methods section, I am still unsure what you mean by "only predictive and in situ observational techniques". How are they different from one another with regards to this manuscript? It appears it is better suited to just say "we aimed to identify spawning periods of Pavona for this region"

**Materials and Methods**

Line 119 | Unnecessary period after Gulf of Thailand

Line 120 | "This was supported by observations" is this citable? If it was personal communication, please state so

Line 138 | Were colonies identified to genus or species-level on the spot via live footage, or identified later while viewing recordings? Please clarify

Line 139 | Very concerned about this medically relevant incident but I think you can just say "growing observations of the *Chironex* sp. box jellyfish (S1 Fig)".

Lines 146-147 | Is there a list of taxa included in each "null data" point? Table S1 suggests that each observation time point for each location is consolidated into one "null data", however, is it "null" because the taxon (e.g. *Pavona explanulata*) was not found in a given location, or a colony was found but it was not spawning? If there is a list of taxa for each time point at a given location, Table S1 should reflect this as it is valuable information.

Lines 147-149 | What was the result of only giving small credence to this "external" dataset? This sentence is a little unclear so either remove, or give a brief example of how the data was treated. e.g. "If the only observation from the site was from an external observer, the spawning period of the given site was treated as inconclusive"

Lines 158-159 | "according to World Register of Marine Species (WoRMS)" instead of "according to the WoRMS standard"

Line 163 | Remove "established"

Line 169 | "broader survey regime" does this refer to something specific that was mentioned before? Please clarify

Lines 174-189 | Under the heading Data analysis, there are a fair bit of parentheses which I suggest removing for readability. For example, sentence starting at line 177 can be turned into a semicolon-separated list starting with "For all data including spawning observations from present study and previous studies compiled, and null data, the following information was collected where relevant: A; B; and C." Another example: the parentheses for line 187 syzygy is unnecessary as the term has been defined in lines 185-186.

**Results**

Throughout | If using a 24-hour clock, "pm" is unnecessary. Please check throughout the manuscript (including in Discussion where a 12-hour clock was used).

Figure 1 and throughout | Please use higher resolution figures, as currently for figure 1, I could barely make out the species names and the black section of the pie chart that indicates spawning duration (e.g. I cannot see a black pie for Koh Larn, *Dipsastraea* sp. in Hin Ploeng, Koh Rangka Jeaw, *Pavona explanulata* in Sail Rock etc., but please see comments below on Figure 1 specifically).

Line 198 | Change from (Fig. 2) to (Fig. 2G)

Line 200 | Table S1 observation No.49 suggests that *P. explanulata* started spawning at 13:12 in Koh Koun, Cambodia. Please clarify.

Lines 214-216 | It is not clear whether the whole sentence is referring to the black portion of the pie only, or the other coloured pies as well. I suggest starting the sentence with "Black pie indicates..." if the sentence is only referring to the black portion of the pie.

Figure 1 | (1) Relating to the comment on Lines 214-216 above, it is not clear what the coloured piece of the pie chart is indicating besides what species it is representing. Currently it could be interpreted as "time since sunrise to onset of spawning", however this should be unnecessary if the black pie is shown. I suggest either colouring in the whole circle to indicate species or change to a simple text label for each species.

(2) Is there a reason the black pie is not shown for some locations/species? If so, please clarify in the figure caption. Looking at Table S1, it seems that in some cases the black pie is not shown in Figure 1 even there were spawning start and end times recorded because there was only one observation? (e.g. Koh Larn, *Pavona varians*) If there was a minimum threshold to make it into the pie charts, please clarify in the figure caption.

(3) My apologies as I am not familiar with the location names, but I cannot see data from "Koh Rangka Jeaw" in S1 Table. Is data for this location under a different name? Also I see that there are a fair few *Pavona* daytime observations from "Hin Yuan" in S1 Table but they are not apparent on Figure 1. Please clarify why some locations were chosen to be shown over others.

Lines 221-222 | What does the red square in Fig. 2B indicate? Is this where Fig. 2A was cropped out of? Please clarify in the figure caption. I am assuming this square was added onto the image later. If not, please ignore this comment.

Line 234 | Does this line correspond to Fig. 2C image? If so, please add (Fig. 2C) after "... confused for sperm release".

Line 246 | Same as Line 234, is this explaining the Fig. 2C image? Also suggest choosing either translucent or transparent

Line 247 | Suggest "...all other daytime spawning non-agariciid corals" and remove the parentheses.

LInes 253-254 | Suggest spelling out the rest of the *Platygyra* trend, e.g. spawned between 102 to 160 hours after lunar peak

Lines 254-256 | Suggest adding the conversion to days in the text or changing the scale in Figure 4 to hours instead of days for consistency

Lines 256-260 & Figures 1&4 | (1) Would it be possible to add the province labels to the map in Figure 1? Reading the lines 256-260 and looking at Figure 4, it is not immediately clear where the boundaries are between provinces and islands, and it will help those of us not from the region.

(2) It seems in Figs. 4C and 4D, the blue dots for Chumphon are at least 30 days apart from the rest or quite a difference between -15 days vs ~-2 days from full moon. Unless there is another dot inside the cloud that I cannot see, I believe this qualifies as a distinct pattern. So I suggest either adding "... however, differences were observed between eastern and western Gulf" after the "No discernible differentiation was observed..." sentence, OR change the first sentence to "No consistent geographic pattern was observed ... except *Pavona* in Chumphon region spawned a month apart from the rest".

Figure 4 | Suggest making the text sizes on the axis titles and labels larger as there seems to be enough space in the plots.

Figure 6 | Similar to comments for Figure 1, the coloured pie is misleading as is, if I am understanding this correctly. The coloured pies currently can be interpreted as spawning has been observed since 0 hours after sunrise for every species and location, as opposed to what you have reported in lines 280-283 "at least 12 hours after sunrise". If using this figure format, each coloured pie should start where the earliest spawning time has been recorded, not at the top of the clock at 0 hours since sunrise. And according to the lines 292-295 figure caption, because Figure 6 is only showing the onset of spawning and not the whole duration of spawning, I believe each circle shouldn't be a pie, and instead should be a single line (or a thinner black pie like in Figure 1 to show variation in start times).

**Discussion**

Lines 300-303 | Although an important aspect, I believe this sentence takes away from the key finding of this paper, which I understood to be the overall coral spawning pattern in the Gulf with particular focus on two daytime spawning *Pavona* species. I believe this section under the heading "Synchrony in the Gulf of Thailand" should be separated into two, and thus, reorganise text accordingly: one, to describe in detail the spawning synchrony observed among species and comparison with previous literature from the region; and another under a heading such as "Non-invasive methods to assess spawning synchrony" to discuss pros and cons of the method.

Lines 317-320 | See comment in the Results section lines 256-260. I believe the lines here should be in the Results section, then further discussed in the Discussion (like you have in lines 320 and onwards).

Line 329 | ...was found "to" differ by

Lines 334-342 | I understood that non-invasive method has limitations, but I am not sure I understand what the rest of the paragraph means. Are you suggesting that based on previous species abundance data, there should be more spawning information on the most abundant taxa e.g. *Astreopora*, *Leptastrea*, *Pavona*, but there isn't any in the literature? And that this present study also did not observe any spawning for those abundant taxa? If this is the case, I suggest spelling that out; for example, "Based on previous biodiversity surveys, we had expected to observe spawning of the two most abundant groups *Leptastrea* and foliose *Pavona*. However, throughout the 5-year survey in present study no spawning was observed for these groups". Additionally, if the focus here is only Agariciidae, perhaps remove *Astreopora* from the list of genera.

Line 357 | Suggest a heading that is a little more descriptive. Since this section only talks about *Pavona* corals, I suggest something along the lines of "Global spawning patterns of *Pavona*"

Lines 372-381 | (1) Parts of this paragraph should be in the Results section, as the only text about the regional differences in spawning time within the year I found was at line 275 "...yet latest in the year", and line 281 "... spawning across various times of the year".

(2) I suggest breaking this part into two parts for readability: one discussing the annual pattern differences (January-May & June spawning in Panama & Hawaii vs September-December spawning in Gulf of Thailand vs Red Sea); and two the differences or lack thereof within the lunar cycle (spawning concentrated around fullmoon to 4 days after). Also I'm not sure what you mean in the parentheses "(with all records between N22° and N7°)", please clarify.

(3) In line 379 "more heavily influenced", this implies that this idea was exclusively tested e.g. with synchrony index (see Bouwmeester et al. 2021 Coral Reefs). Perhaps the wording can be changed to highlight that the observed pattern **coincided** with longitudinal separations. However, for exploratory purposes I suggest you at least mention other possible factors such as temperature regimes and taxonomic uncertainties just like in the introduction lines 58-59. I can see that light regimes and taxonomic uncertainties are discussed more later under "Broader Insights", so perhaps only list possible factors here, then write "see below".

LIne 384 | Was this meant to be a citation (in [number] format) or a typo for "from Taiwan by Lin and Nozawa [57]"?

Line 393 | Remove apostrophe from "author's"

Line 408 | Possible typo of "recorded as"?

Line 409 | Change "spawning after sunset " to "spawn after sunset"

Line 412-413 | (1) I believe these *Pavona* spp. are the ones referred to as the foliose *Pavona* at lines 334-342. This information "(abundant across the Gulf of Thailand)" should be presented earlier if this is the case.

(2) Was *P. frondifera* recorded in this present study? I can only see *P. decussata* in Figure 5B. If the claim of *P. frondifera* being a nighttime spawner is based on null data, that distinction is necessary.

Line 414 | Suggest "Across the genus-level" instead of "When looked at in aggregate"

Lines 415-417 | Suggest breaking this sentence into at least two for readability.

Line 417 | "correlated"

Line 424 | Suggest a more descriptive heading. Perhaps add "into daytime spawning".

Lines 431-432 | Which description? In the methods section lines 156-158, citations indicate that series of recent literature was used, not the original description by Verrill 1864.

Lines 429-436 | I believe there are two ideas presented here. One, that your data from Gulf of Thailand identified *Pavona* species using multiple lines of evidence, so adding confidence that each *Pavona* species are good distinct species. The second idea is that the global dataset of *Pavona,* for example the putatively widely distributed *P. varians* may contain multiple species. This is also valid because three nominal species are currently junior synonyms of *P. varians* (Verrill, 1864): *P. repens* (Brüggemann, 1877), *P. intermedia* (Gardiner, 1898), and *P. percarinata* (Ridley, 1883). If you look at the original description of each species, you can see that they are all described from different locations. And like it says at the end of the paragraph (lines 435-436), molecular techniques may also reveal that there are other undescribed species, so it is possible that the regional differences are due to "*P. varians*" containing multiple species. In short, I suggest reorganising this paragraph so that it highlights that (1) the global dataset of one "widely distributed species" may contain multiple species, and (2) each of your study taxon is identified as distinct species unit based on multiple lines of evidence, so it is a valid dataset no matter what the identity of the organisms may be.

Having said that, if you do reorganise this paragraph this way, I suggest including images of the representatives of key species as supplementary material (just like Figure 2). That way there is a better chance of future proofing your data in case of taxonomic updates.

Lines 442-444 | I expected to see a longer list of citations with a sentence starting "There is no shortage of literature". Suggest adding relevant literature that is already in the reference list (such as the same ones from the Introduction). Also suggest "nighttime" spawning time here.

Lines 468-469 | Perhaps add "earlier" before daytime spawning? Other Pavonas from the global dataset also spawns during daytime.

Paragraph between lines 463-488 | It seems that there are several ideas presented in this paragraph which makes it difficult to comprehend. The opening sentence suggests that photoprotective mechanisms may differ between nighttime and daytime spawners, but the following sentences do not compare known mechanism in typical nighttime spawners vs daytime spawners. I suggest re-writing this paragraph for clarity.

Lines 489-493 | This should go in the introduction, around lines 74-78. Then the sentence can simply start with "Our data contributes to the growing observation of daytime spawning in scleractinian corals. In particular, ..."

Lines 506-508 | This sentence contain 43 words. I would remove unnecessary words such as "certainly", and separate this into at least two sentences e.g. "Further investigation is needed for the geographical extent of daytime *Pavona* spawning. Questions also arise whether fertilisation and recruitment remain viable under UV exposure ..."

Line 515 | due "to"

Lines 529-534 | This seems out of place and while interesting, rather irrelevant to the manuscript. Perhaps keep it in the supplementary or a separate observation note?
